# Temporal and tissue-specific requirements for T-lymphocyte IL-6 signalling in obesity-associated inflammation and insulin resistance

**DOI:** 10.1038/ncomms14803

**Published:** 2017-05-03

**Authors:** Elaine Xu, Mafalda M. A. Pereira, Ismene Karakasilioti, Sebastian Theurich, Mona Al-Maarri, Gunter Rappl, Ari Waisman, F. Thomas Wunderlich, Jens C. Brüning

**Affiliations:** 1 Max Planck Institute for Metabolism Research, Department of Neuronal Control of Metabolism, 50931 Cologne, Germany; 2 Center for Endocrinology, Diabetes and Preventive Medicine (CEDP), University Hospital, 50924 Cologne, Germany; 3 Excellence Cluster on Cellular Stress Responses in Aging Associated Diseases (CECAD) and Center of Molecular Medicine Cologne (CMMC), University of Cologne, 50931 Cologne, Germany; 4 Center for Molecular Medicine Cologne (CMMC) and Department of Internal Medicine I, University of Cologne, 50931 Cologne, Germany; 5 Institute for Molecular Medicine, University of Medical Centre of the Johannes Gutenberg University of Mainz, 55131 Mainz, Germany; 6 National Center for Diabetes Research (DZD), 85764 Neuherberg, Germany

## Abstract

Low-grade inflammation links obesity to insulin resistance through the activation of tissue-infiltrating immune cells. Interleukin-6 (IL-6) is a crucial regulator of T cells and is increased in obesity. Here we report that classical IL-6 signalling in T cells promotes inflammation and insulin resistance during the first 8 weeks on a high-fat diet (HFD), but becomes dispensable at later stages (after 16 weeks). Mice with T cell-specific deficiency of IL-6 receptor-α (IL-6Rα^T-KO^) exposed to a HFD display improved glucose tolerance, insulin sensitivity and inflammation in liver and EWAT after 8 weeks. However, after 16 weeks, insulin resistance in IL-6Rα^T-KO^ epididymal white adipose tissue (EWAT) is comparable to that of controls, whereas the inflammatory profile is significantly worse. This coincided with a shift from classical T cell IL-6 signalling at 8 weeks, to enhanced IL-6 trans-signalling at 16 weeks. Collectively, our studies reveal that IL-6 action in T cells through classical IL-6 signalling promotes inflammation and insulin resistance early during obesity development, which can be compensated for by enhanced IL-6 trans-signalling at later stages.

## Abstract (web summary)

Interleukin-6 (IL-6) is increased in obesity and activates T cells to promote inflammation. Here, Xu *et al*. use mice that lack IL-6 receptors on T cells to uncover the temporal and tissue-specific effects of classic and trans IL-6 signalling on inflammation and insulin resistance on a high-fat diet.

Chronic and low-grade inflammation in insulin target tissues is tightly associated with insulin resistance^1^,^2^,^3^,^4^. The development of obesity-induced inflammation is orchestrated by resident and infiltrating immune cells in a synchronized and timed manner^2^. Macrophages and related dendritic cells are primarily responsible for the onset and the maintenance of tissue inflammation^5^. Infiltration of these cells can induce and/or be elicited by the sequential changes in the composition of different T-lymphocytes^6^,^7^ while T cells in visceral white adipose tissue also directly contribute to the proinflammatory microenvironment^8^. In particular, the CD4+ T helper (Th) cell types, Th1 and Th17, identified by their specific secretion of interferon-γ (IFNγ) and interleukin (IL)-17, respectively, promote obesity-associated tissue inflammation^9^,^10^,^11^,^12^,^13^. In obese humans, both CD8+ and CD4+ T cells, specifically the Th1, Th2 and Th17 cell populations, in both visceral and subcutaneous white adipose tissue are associated with systemic inflammation and insulin resistance^14^,^15^.

Among the immune-modulating cytokines dysregulated in obesity, IL-6 is one of the most frequently implicated cytokine, as its elevated circulating levels are consistently observed in obese mouse models and humans^16^,^17^. Owning to a broad spectrum of biological activities, IL-6 is also an important regulator of T cells. By protecting T cells from apoptosis, IL-6 signals to promote T cell development^18^ especially for CD4+ Th cells^19^. During the propagation of immune responses, IL-6 promotes the differentiation of naive T cells into Th cells^20^. In acute inflammation, IL-6 is also responsible for T cell activation, tissue infiltration and memory maintenance^21^,^22^. In addition, IL-6 is required for effector T cells to overcome the suppression by regulatory T cells (Treg)^22^,^23^, while inhibiting the differentiation of naive CD4+ T cells into Treg^24^. Since immunotherapy targeting T cells normalizes glucose homeostasis^9^, and as T cell inhibitors reduce CD8+ T cells and proinflammatory macrophages in visceral adipose tissue^25^, we investigated whether abrogating IL-6 signalling in T cells would affect the development of obesity-associated tissue inflammation and, subsequently, alter systemic glucose homeostasis.

We generated T cell-specific IL-6Rα knockout mice (IL-6Rα^T-KO^) and subjected them to diet-induced obesity via exposure to a high-fat diet (HFD, 60% Kcal fat) for 8 and 16 weeks, at which points their metabolic phenotype was characterized and the concurrent inflammatory state of liver and epididymal white adipose tissue (EWAT) was assessed. After 8 weeks of HFD feeding, IL-6Rα^T-KO^ mice display an improved overall metabolic and inflammatory phenotype compared with littermate controls. Interestingly, prolonged HFD feeding (16 weeks) renders the IL-6Rα^T-KO^ EWAT more inflamed than that of IL-6Rα^f/f^ controls. At this point, IL-6Rα^T-KO^ animals harbour glucose and insulin similar to their littermate controls tolerance and perform significantly worse during the hyperinsulinaemic-euglycaemic (HIEG) clamp experiments. This results from normalized IL-6 signalling via the soluble IL-6 receptor-α (sIL-6Rα) in the IL-6Rα-deficient T cells, as both IL-6 and sIL-6Rα levels as well as the intrinsic responsiveness of T cell to IL-6 trans-signalling were significantly elevated. Thus, our data demonstrate differential temporal and tissue-specific functions of IL-6 signalling in T-lymphocytes, as well as the time-dependent importance of the classical and trans-signalling of IL-6 during the development of obesity-associated inflammation and insulin resistance.

## Results

### Improved glucose homeostasis in young obese IL-6Rα^T-KO^ mice

To generate mice with T cell-specific IL-6Rα deficiency (IL-6Rα^T-KO^), we crossed mice hemizygous for a transgene in which transcription of the Cre recombinase is controlled by the *Cd4* promoter (CD4-Cre)^26^ with mice homozygous for *LoxP*-flanked *Il6ra* alleles (IL-6Rα^f/f^)^27^. As the CD4 gene is expressed in thymocytes during T cell differentiation from CD4−CD8− to the CD4+CD8+ stage^26^, both CD4+ and CD8+ mature T cells were deficient in IL-6Rα (Supplementary Fig. 1a) at both 8 and 16 weeks of HFD feeding, despite the generally suppressed expression of IL-6Rα at 16 weeks (Supplementary Fig. 1b). The Cre-mediated excision of *loxP*-flanked *Il6ra* in T-lymphocytes was confirmed both via PCR analysis of genomic DNA and flow cytometry analyses of IL-6Rα (Supplementary Fig. 1c,d), while the expression of IL-6Rα expectedly remained intact in CD11c+ cells (Supplementary Fig. 1d).

**Figure 1:**
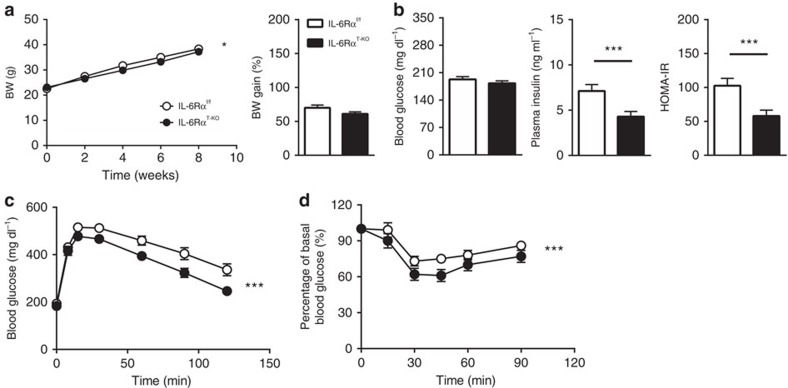
Physiological parameters and metabolic characterization of IL-6Rα^T-KO^ mice (8-week HFD). (**a**) Body weight (BW) curve on HFD and percentage of body weight gain (*n*=21 versus 26). (**b**) Fasting (6 h) blood glucose (*n*=21 versus 26), plasma insulin and HOMA-IR (*n*=18 versus 16). (**c**) Blood glucose concentrations during intraperitoneal glucose tolerance tests (IPGTT) after a 6 h fast (*n*=21 versus 26). (**d**) Blood glucose as a percentage of basal value during intraperitoneal insulin tolerance tests (IPITT) after a 2 h fast (*n*=15 versus 18). Two-tailed *t*-tests and two-way analysis of variance (ANOVA) used for statistical analyses (*P<0.05; ***P<0.005). Error bars presented as s.e.m.

To initiate diet-induced obesity, we subjected male IL-6Rα^T-KO^ animals to the HFD regimen with their IL-6Rα^f/f^ littermates as controls at 8 weeks of age, when IL-6Rα^T-KO^ mice showed similar body weight as the IL-6Rα^f/f^ controls (Fig. 1a). Over a period of 8 weeks of HFD feeding, the body weight of both groups increased, though slightly but significantly less for IL-6Rα^T-KO^ mice (Fig. 1a). Although after daytime fasting of 6 h the blood glucose did not differ between the two genotypes, plasma insulin and calculated homeostasis model assessment of insulin resistance (HOMA-IR) of IL-6Rα^T-KO^ animals were significantly lower than that of controls (Fig. 1b), indicating an improved glucose homeostasis during the fasting state. IL-6Rα^T-KO^ mice also performed better during intraperitoneal glucose and insulin tolerance tests (Fig. 1c,d), displaying more sensitivity of metabolic tissues to endogenous and exogenous insulin. At this time point, IL-6Rα^T-KO^ mice showed no alteration in insulin secretion or clearance during the dynamic intravenous glucose-stimulated insulin secretion test, except the lower blood glucose levels throughout (Supplementary Fig. 2a–d), which was consistent with data from the intraperitoneal glucose tolerance test.

**Figure 2:**
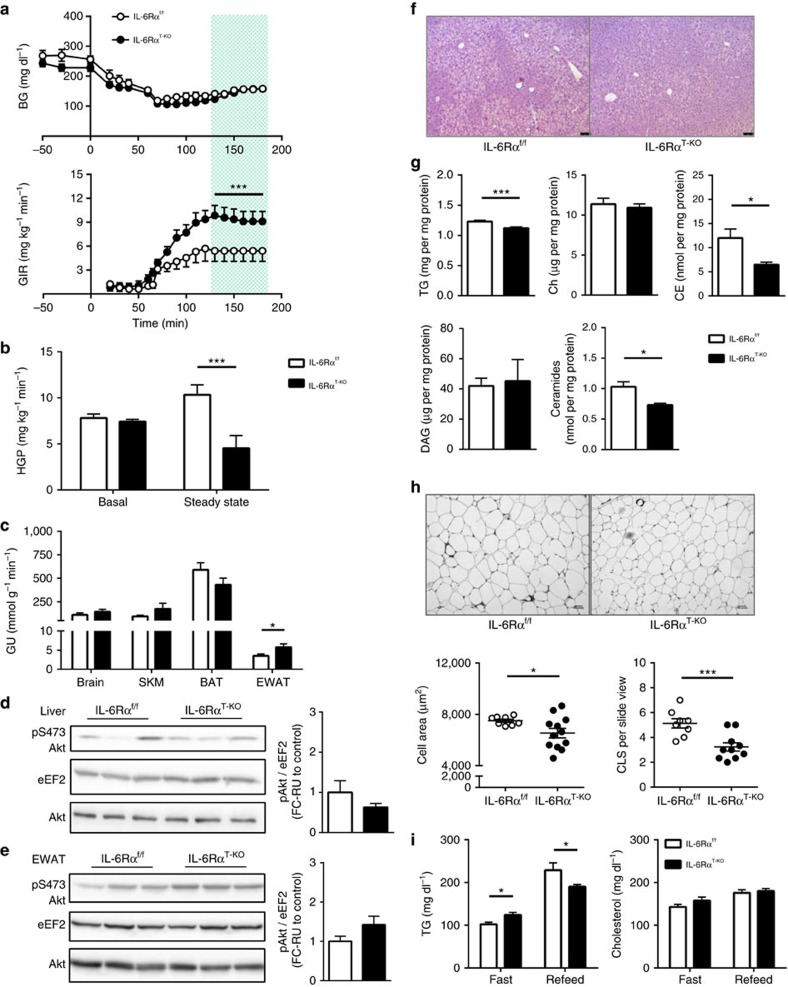
Improved glucose homeostasis and lipid metabolism in IL-6Rα^T-KO^ (8-week HFD). (**a**) Blood glucose (BG) and glucose infusion rate (GIR) during hyperinsulinaemic-euglycaemic (HIEG) clamp experiments (*n*=9); two-way analysis of variance (ANOVA) was performed on data from the green-shaded steady state (130–180 min). (**b**) Rate of hepatic glucose production (HGP) at basal and during steady state of HIEG clamp (*n*=9). (**c**) Rate of glucose uptake (GU) during steady state of HIEG clamp (*n*=9). Representative immunoblots of 3 blots and quantification of (**d**) liver and (**e**) EWAT pS473 Akt, total Akt and eEF2 at time 180 min during steady state of HIEG clamp (*n*=9); relative unit (RU) to that of IL-6Rα^f/f^ (Control). (**f**) Representative liver sections of 22 samples with haematoxylin and eosin (H&E) staining; scale bars, 75 μm. (**g**) Hepatic content of triglycerides (TG), free cholesterols (Ch), cholesteryl esters (CE), diglycerides (DAG) and ceramides (*n*=6). (**h**) Representative EWAT sections of 20 samples with F4/80 staining and quantification of adipocyte size in area and crown-like structure (CLS) counts (*n*=8 versus 12); scale bars, 100 pixels. (**i**) Fast (12 h) and refeed (2 h) plasma TG and cholesterol levels (*n*=19 versus 20). Two-tailed *t*-tests and two-way ANOVA used for statistical analyses (*P<0.05; ***P<0.005). Error bars presented as s.e.m.

To further evaluate insulin action in these animals, HIEG clamp studies were conducted^28^. As blood glucose was being titrated to ∼140 mg dl^−1^, the infusion rate of glucose solution to achieve and maintain this euglycaemia for IL-6Rα^T-KO^ mice was significantly higher than for IL-6Rα^f/f^ controls (Fig. 2a), supporting an improvement in systemic insulin sensitivity in these animals. With similar basal rates of hepatic glucose production (HGP), the steady-state HGP was significantly decreased in IL-6Rα^T-KO^ mice, demonstrating an improved ability of insulin to suppress glucose output in these animals (Fig. 2b). Insulin-stimulated tissue glucose uptake as measured by ^14^C-labelled deoxyglucose uptake during the HIEG experiment revealed a significant increase in the EWAT of IL-6Rα^T-KO^ animals compared with that of IL-6Rα^f/f^ controls, while the rate of glucose uptake was similar in brain, skeletal muscles and brown adipose tissue (Fig. 2c). These changes occurred in the absence of any differences in total circulating levels of insulin or endogenous insulin secretion during the HIEG clamps (Supplementary Fig. 3a,b). Collectively, these data indicate that the previously detected improvement of systemic insulin sensitivity in IL-6Rα^T-KO^ mice was a result of improved insulin action in liver and EWAT.

Next, western blotting for pAkt on the serine 473 residue was performed on liver and EWAT of clamped control and IL-6Rα^T-KO^ mice to monitor insulin signalling at the molecular level. Insulin-stimulated phosphorylation of hepatic Akt was comparable between IL-6Rα^f/f^ and IL-6Rα^T-KO^ animals, with a tendency of higher activation of Akt phosphorylation in the EWAT of IL-6Rα^T-KO^ mice than that observed in controls (Fig. 2d,e), providing support for the detected increase in the insulin-stimulated glucose uptake in the EWAT of IL-6Rα^T-KO^ animals.

To verify the basic metabolic parameters, indirect calorimetric measurements were performed with animals at 14 weeks of HFD feeding from three separate cohorts. Cumulative food intake, water intake, activity, average energy expenditure and respiratory quotient were comparable between the two genotypes of mice (Supplementary Fig. 4a–e).

### Improved lipid homeostasis in young obese IL-6Rα^T-KO^ mice

Although lipid accretion in the liver does not always occur simultaneously with hepatic insulin resistance^29^,^30^,^31^,^32^, they often correlate in diet-induced obesity^33^,^34^,^35^. Being exposed to HFD for 8 weeks, the livers of IL-6Rα^T-KO^ mice weighed less than those from IL-6Rα^f/f^ controls (Supplementary Table 1) and acquired less lipids (Fig. 2f), and particularly the total triglycerides (TGs), cholesteryl esters and ceramides decreased significantly in these animals (Fig. 2g). This improvement of hepatic steatosis in IL-6Rα^T-KO^ animals was also paralleled by reduced signs of liver damage, as evidenced by lower serum levels of aspartate aminotransferase and alanine transaminase (Supplementary Table 2).

Unlike the relationship between hepatic lipid accumulation and insulin resistance, unilobular lipid accumulation in adipocytes of visceral adipose tissue, especially the EWAT in mice, potently induces severe local and systemic insulin resistance^36^,^37^,^38^,^39^. The EWAT adipocytes, measured by average cell area, were significantly smaller from IL-6Rα^T-KO^ animals as compared with those from IL-6Rα^f/f^ controls (Fig. 2h), corresponding to the improved insulin sensitivity observed in the experiments described above. To assess the overall plasticity of lipid homeostasis, circulating TG and cholesterol levels were measured after a 12 h fast and at the end of the subsequent 2 h refeeding period of the same HFD. Parallel to the improved glucose homeostasis, the lipid metabolism of IL-6Rα^T-KO^ mice was also more flexible than that in control IL-6Rα^f/f^ animals, as demonstrated by the significantly higher fasting serum TG followed by significantly lower TG levels after refeeding, whereas the serum cholesterol levels were not different (Fig. 2i).

### Reduced inflammation in IL-6Rα^T-KO^ mice after 8 weeks of HFD

**Figure 3:**
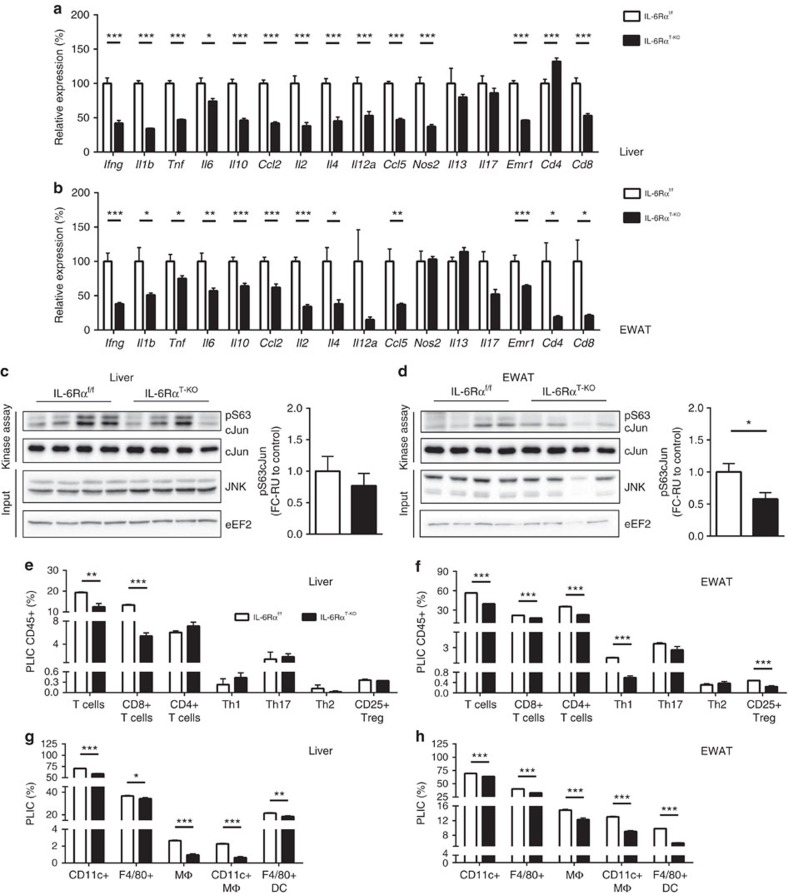
Inflammatory profile of liver and EWAT (8-week HFD). (**a**) Liver (*n*=10 versus 12) and (**b**) EWAT (*n*=11) gene expression profile. Activity of c-Jun kinase (JNK) in (**c**) liver and (**d**) EWAT with the measurement of pS63 c-Jun by immunoblotting (representative immunoblots of 2 blots) and quantification (*n*=8); relative unit (RU) to that of IL-6Rα^f/f^ (Control). Flow cytometry analyses of (**e**) liver and (**f**) EWAT composition of total T cells, CD8+ and CD4+ T cells, T helper cells (Th1, Th17 and Th2) and CD25+ regulatory T cells (Treg), presented as percentage of live immune cells (PLICs) positive for CD45 (*n*=3 analysed samples pooled from *n*=4–8 animals per sample). Flow cytometry analyses of (**g**) liver and (**h**) EWAT composition of total CD11c+ myeloid cells, F4/80+ myeloid cells, total macrophages (MΦ), CD11c+ MΦ and F4/80+ dendritic cells (DCs), presented as PLICs (*n*=3 analysed samples pooled from *n*=4–8 animals per sample). Two-tailed *t*-tests and two-way analysis of variance (ANOVA) used for statistical analyses (*P<0.05; **P<0.01; ***P<0.005). Error bars presented as s.e.m.

To determine whether the improved glucose and lipid metabolism observed in IL-6Rα^T-KO^ mice was due to altered inflammation resulting from the lack of IL-6 action in T cells, we assessed the tissue inflammatory profile in both liver and EWAT of IL-6Rα^T-KO^ and control mice. The most prominent sign of inflammation in white adipose tissue of obese animals and humans are the crown-like structures (CLSs) that comprise infiltrating macrophages around dying or dead adipocytes^40^. In the EWAT of IL-6Rα^T-KO^ animals, the average number of CLSs per tissue section was significantly lower than that in the EWAT of IL-6Rα^f/f^ controls (Fig. 2h), indicating an improved EWAT inflammation in these animals. During the development of tissue inflammation, active immune cells interact with each other and the parenchymal cells of the organ through the secretion of cytokines, creating a dynamic inflammatory microenvironment. The gene expression of major proinflammatory cytokines and chemokines in both liver and EWAT of IL-6Rα^T-KO^ mice was significantly lower than that in control mice (Fig. 3a,b), thereby further supporting the notion of improved inflammation in these mice. In particular, the lower gene expression of IFNγ, specifically secreted from activated Th1 (ref. 11) and cytotoxic CD8+ T cells^7^, of IL-6, produced by most T cells, of IL-2, rapidly released by CD4+ T cells^41^, and of IL-4, the Th2 signature cytokines that protects T helper cells from Treg-induced suppression^42^, insinuates reduced contribution by T cells to tissue inflammation.

Activation of c-Jun N-terminal kinase (JNK) in both brain and peripheral metabolic tissues and in immune cells is a major link between chronic tissue inflammation and insulin resistance in obesity^4^,^43^,^44^,^45^,^46^,^47^. As a result of the amended inflammatory microenvironment in liver and EWAT of IL-6Rα^T-KO^ animals, JNK activity showed a tendency of reduction in the liver (Fig. 3c) and was significantly reduced in the EWAT of IL-6Rα^T-KO^ mice (Fig. 3d).

Since the lack of IL-6Rα signalling in T cells has been found to affect the activation and functions of T cells but not their development^22^, we analysed the immune-cell composition in liver and EWAT by flow cytometry. As expected, we observed a significant reduction in the composition of total T cells and CD8+ T cells within the entire population of live CD45+ immune cells isolated from both liver and EWAT from IL-6Rα^T-KO^ mice compared with those from control animals upon 8 weeks of HFD feeding (Fig. 3e,f). A prominent reduction in CD4+ T cells, particularly the Th1 and Treg, was also detected in the EWAT but not in the liver of IL-6Rα^T-KO^ mice (Fig. 3e,f), suggesting a tissue-specific variation in the infiltration of different T cell subpopulations as a result of abrogated IL-6 action in T cells. Given the reduced gene expression of IFNγ (Fig. 3a,b) and its sources, namely Th1 and CD8+ T cells (Fig. 3e,f), and that IFNγ signalling is essential for macrophage function^48^, less CD11c+ myeloid cells were identified in the liver and EWAT of IL-6Rα^T-KO^ animals compared with those of control mice (Fig. 3g,h). In particular, the percentage of proinflammatory myeloid cells, F4/80+ cells and CD11c+ macrophages was significantly lower in the liver and EWAT of IL-6Rα^T-KO^ mice (Fig. 3g,h). The reduction of F4/80+ CD11+ macrophages in IL-6Rα^T-KO^ EWAT was consistent with the reduced CLS appearance (Fig. 2h) and gene expression of IL-1β, IL-6 and tumour necrosis factor (Fig. 3b). In confirmatory observation, the composition of F4/80+ myeloid cells as well as CD4+ and CD8+ T cells in both liver and EWAT (Fig. 3e–h) was correspondingly reflected by the gene expression of F4/80 (*Emr1*), CD4 and CD8, respectively (Fig. 3a,b).

### Prolonged HFD worsens EWAT inflammation in IL-6Rα^T-KO^ mice

**Figure 4:**
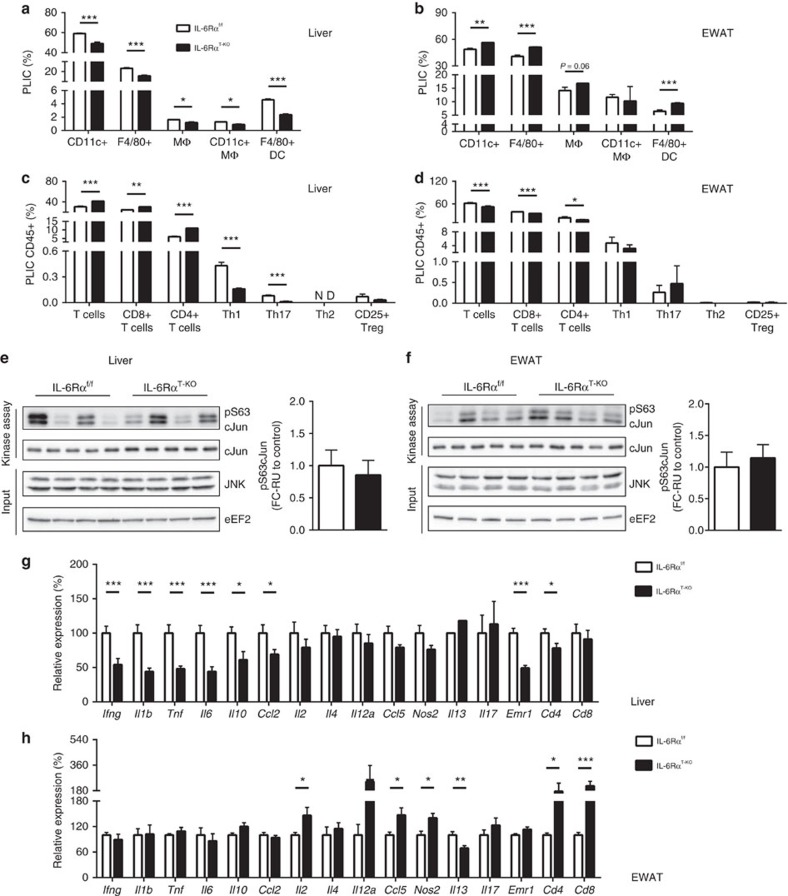
Inflammatory profile of liver and EWAT (16-week HFD). Flow cytometry analyses of (**a**) liver and (**b**) EWAT composition of total CD11c+ myeloid cells, F4/80+ myeloid cells, total MΦ, CD11c+ MΦ and F4/80+ DC, presented as PLICs (*n*=3 analysed samples pooled from *n*=4–8 animals per sample). Flow cytometry analyses of (**c**) liver and (**d**) EWAT composition of total T cells, CD8+ and CD4+ T cells, Th1, Th17, Th2 and CD25+ Treg, presented as PLIC CD45+ (*n*=3–5 analysed samples pooled from *n*=4–8 animals per sample); ND, not detected. JNK activity in (**e**) liver and (**f**) EWAT with the measurement of pS63 c-Jun by immunoblotting (representative immunoblots of 2 blots) and quantification (*n*=8); relative unit (RU) to that of IL-6Rα^f/f^ (Control). (**g**) Liver and (**h**) EWAT gene expression profile (*n*=11). Two-tailed *t*-tests and two-way analysis of variance (ANOVA) used for statistical analyses (*P<0.05; **P<0.01; ***P<0.005). Error bars presented as s.e.m.

To evaluate the effect of T cell IL-6Rα deficiency in the development of obesity-associated chronic inflammation, we subjected IL-6Rα^T-KO^ mice and their IL-6Rα^f/f^ littermates to a prolonged HFD exposure of 16 weeks. To our surprise, while the liver of IL-6Rα^T-KO^ mice was still protected from massive infiltration of proinflammatory myeloid cells (Fig. 4a), their EWAT was burdened with significantly more CD11c+ and F4/80+ cells compared with that of control mice upon prolonged HFD feeding (Fig. 4b). Compared with the T-lymphocyte composition in the liver and EWAT of IL-6Rα^f/f^ controls, significantly less proinflammatory Th1 and Th17 populations, though more CD4+, CD8+ and total T cells, were found in the liver of IL-6Rα^T-KO^ animals (Fig. 4c). No alteration of specific T helper cells but a slight difference in CD4+, CD8+ and total T cells were detected in the EWAT of IL-6Rα^T-KO^ mice at this point (Fig. 4d). These flow cytometry data revealed more immune cells with proinflammatory properties in the EWAT and less in the liver of IL-6Rα^T-KO^ animals, unravelling a more clear differentiation of infiltrating immune cells between liver and visceral white adipose tissue with prolonged HFD feeding as compared with the data at 8 weeks of HFD feeding (Fig. 3e–h). Concurrently, JNK activity showed no major difference in the liver of IL-6Rα^T-KO^ mice (Fig. 4e) or in their EWAT (Fig. 4f) compared with that in control animals.

The gene expression of various cytokines and chemokines that was reduced in the liver of IL-6Rα^T-KO^ mice at 8 weeks of HFD feeding became similar to that in controls after 16 weeks of HFD feeding, while the gene expression of the proinflammatory cytokines and chemokines, IFNγ, IL-1β, tumour necrosis factor, IL-6 and monocyte chemoattractant protein 1 (MCP1; *Ccl2*) remained significantly improved even upon prolonged HFD feeding (Fig. 4g). In contrast, in the EWAT of IL-6Rα^T-KO^ mice, the overall improvement of inflammatory gene expression observed at 8 weeks of HFD feeding was completely abolished at this prolonged time point (Fig. 4h). In addition, the gene expression of IL-2, IL-12a, regulated on activation, normal T cell expressed and secreted (RANTES; *Ccl5*) and inducible nitric oxide synthase (iNOS; *Nos2*) was even significantly higher at this point than that in the EWAT of control animals (Fig. 4h).

### Prolonged HFD renders IL-6Rα^T-KO^ mice insulin resistant

**Figure 5:**
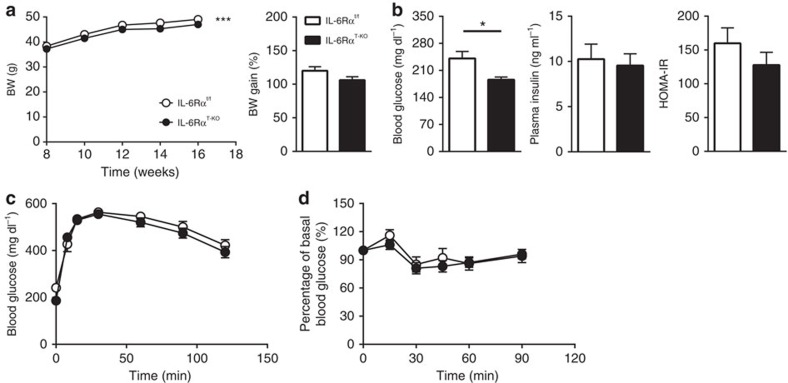
Physiological parameters and metabolic characterization of IL-6Rα^T-KO^ mice (16-week HFD). (**a**) BW curve from 8 to 16 weeks on HFD and percentage of body weight gain (*n*=22 versus 25). (**b**) Fasting (6 h) blood glucose (*n*=22 versus 25), plasma insulin and HOMA-IR (*n*=16 versus 19). (**c**) Blood glucose concentrations during IPGTT after a 6-h fast (*n*=22 versus 25). (**d**) Blood glucose as a percentage of basal value during IPITT after a 2-h fast (*n*=16 versus 21). Two-tailed *t*-tests and two-way analysis of variance (ANOVA) used for statistical analyses (*P<0.05; ***P<0.005). Error bars presented as s.e.m.

During the extra 8 weeks of prolonged HFD feeding, the body weight of both groups continued to increase, and the IL-6Rα^T-KO^ animals continued to weigh slightly less than their controls (Fig. 5a). At 24 weeks of age and after 16 weeks of HFD feeding, the fasting blood glucose of IL-6Rα^T-KO^ mice was improved, but the fasting plasma insulin and calculated HOMA-IR became comparable to that of control mice (Fig. 5b). At this point, glucose and insulin tolerance were also indistinguishable between IL-6Rα^T-KO^ and control mice (Fig. 5c,d). Although IL-6Rα^T-KO^ animals showed a modest improvement in glucose tolerance with comparable plasma insulin levels as control IL-6Rα^f/f^ mice following an intravenous administration of glucose, the clearance of insulin, as reflected by the ratio of insulin to C-peptide, was significantly delayed (Supplementary Fig. 5a–d), indicating the presence of hepatic insulin resistance^49^.

**Figure 6:**
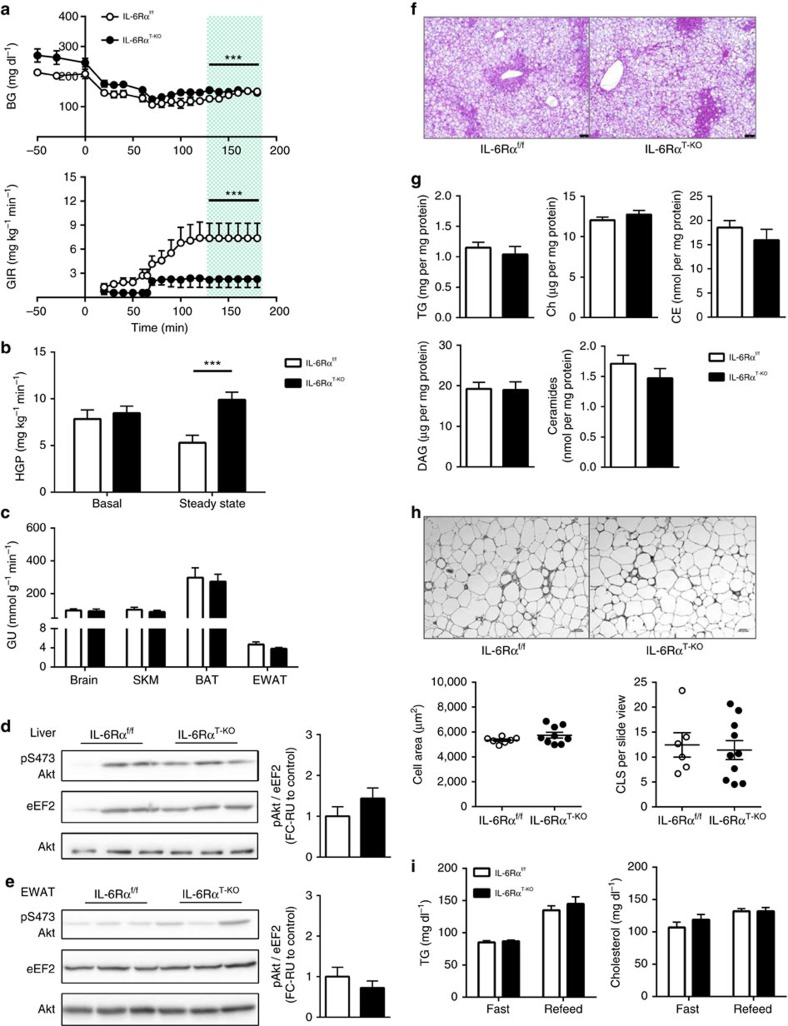
Severe glucose homeostasis and lipid metabolism in IL-6Rα^T-KO^ (16-week HFD). (**a**) BG and GIR during HIEG clamp experiments (*n*=6 versus 9); two-way analysis of variance (ANOVA) was performed on data from the green-shaded steady state (130–180 min). (**b**) Rate of HGP at basal and during steady state of HIEG clamp (*n*=6 versus 9). (**c**) Rate of GU during steady state of HIEG clamp (*n*=6 versus 9). Representative immunoblots of 3 blots and quantification of (**d**) liver and (**e**) EWAT pS473 Akt, total Akt and eEF2 at time 180 min during steady state of HIEG clamp (*n*=6 versus 9); relative unit (RU) to that of IL-6Rα^f/f^ (Control). (**f**) Representative liver sections of 21 samples with haematoxylin and eosin (H&E) staining; scale bars, 75 μm. (**g**) Hepatic content of TG, Ch, CE, DAG and ceramides (*n*=9 versus 10). (**h**) Representative EWAT sections of 16 samples with F4/80 staining and quantification of adipocyte size in area and CLS counts (*n*=7 versus 9); scale bars, 100 pixels. (**i**) Fast (12 h) and refeed (2 h) plasma TG and cholesterol levels (*n*=16 versus 17). Two-tailed *t*-tests and two-way ANOVA used for statistical analyses (***P<0.005). Error bars presented as s.e.m.

During HIEG clamp studies, a minimal rate of glucose infusion was required in IL-6Rα^T-KO^ mice to maintain euglycaemia (Fig. 6a), showing further deterioration of systemic insulin sensitivity upon prolonged HFD feeding. With a similar rate of basal HGP, the steady-state HGP during the HIEG clamp was significantly increased in IL-6Rα^T-KO^ animals, demonstrating an impaired ability to suppress HGP in these mice (Fig. 6b). With higher total circulating levels of insulin and unaltered endogenous insulin secretion (Supplementary Fig. 6a,b), insulin-stimulated glucose uptake in all investigated tissues of IL-6Rα^T-KO^ mice was comparable to that in controls (Fig. 6c). Collectively, these results point to a time- and diet-dependent exacerbation of insulin resistance in IL-6Rα^T-KO^ mice. Consistent with a progressive deterioration of insulin sensitivity upon prolonged HFD feeding in IL-6Rα^T-KO^ mice, the insulin-stimulated activation of tissue Akt, as detected by pS473 Akt, became comparable between IL-6Rα^T-KO^ and control mice (Fig. 6d,e).

Following 8 additional weeks of HFD feeding, the livers of IL-6Rα^T-KO^ mice weighed the same as those of control mice (Supplementary Table 3) and showed a similar degree of hepatic steatosis (Fig. 6f). With comparable contents of TG, free cholesterols, cholesteryl esters, diglycerides and ceramides as those in the livers of control mice (Fig. 6g), the livers of IL-6Rα^T-KO^ animals exhibited even more tissue damage, as reflected by higher serum levels of aspartate aminotransferase (Supplementary Table 4). These findings reflected a complete loss of protection against ectopic hepatic fat accumulation.

In the EWAT of IL-6Rα^T-KO^ mice, adipocytes have expanded to be as large as those in the EWAT of control animals, and the tissue infested with comparable amount of CLS (Fig. 6h), correlating to the rapidly exacerbated inflammation upon prolonged HFD feeding in these mice. Similarly, the overall improvement of lipid homeostasis as revealed by the regulatory flexibility of circulating TG levels previously observed at 8 weeks of HFD feeding was completely lost (Fig. 6i).

### IL-6 trans-signalling in IL-6Rα^T-KO^ mice after prolonged HFD

Although IL-6 signalling via its classic transmembrane IL-6Rα was abolished in the T cells of IL-6Rα^T-KO^ mice, IL-6 could still bind to the sIL-6Rα with similar affinity^50^ as a complex that signals through the intact gp130 on the IL-6Rα-deficient T cells. Upon various stimuli, shedding generates sIL-6Rα^51^,^52^,^53^ via cleavage of the transmembrane IL-6Rα by ADAM17 (ref. 54) and ADAM10 (ref. 55) on many cells expressing normal IL-6Rα, and sIL-6Rα can be found in circulation, urine and almost all tissues to exert its proinflammatory effects^52^,^53^,^56^.

**Figure 7:**
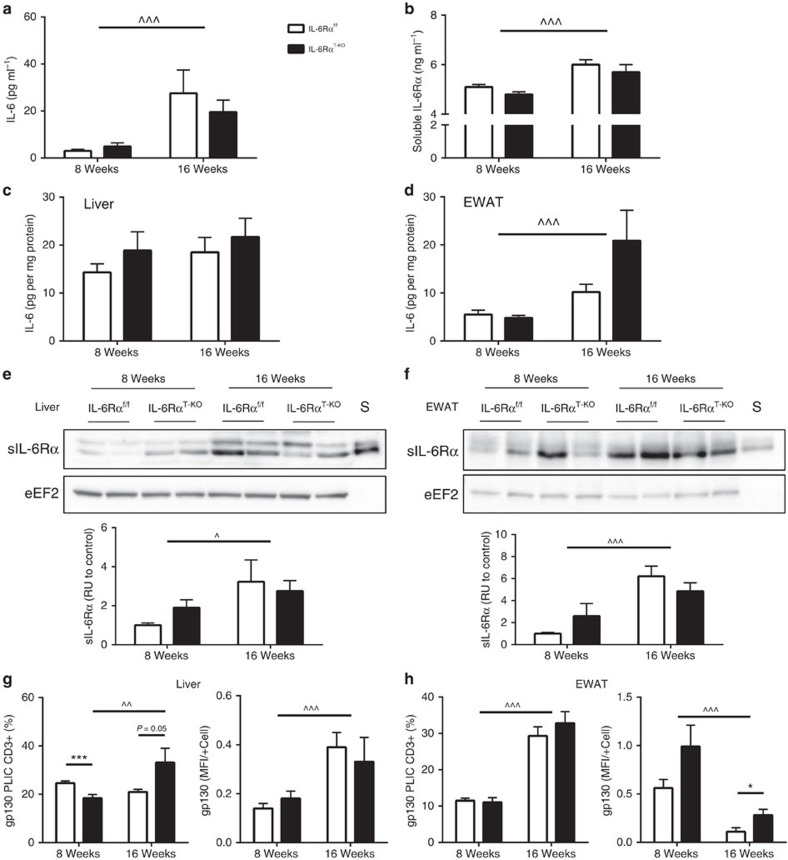
Increased IL-6 and sIL-6Rα levels upon prolonged HFD feeding (8- and 16-week HFD). (**a**) Serum IL-6 levels (*n*=11 versus 10–14). (**b**) Serum sIL-6Rα levels (*n*=20 versus 20). (**c**) Liver IL-6 content (*n*=11–12 versus 11–12). (**d**) EWAT IL-6 content (*n*=11–12 versus 11–12). Representative immunoblots and quantification of (**e**) liver (*n*=8 versus 8) and (**f**) EWAT sIL-6Rα and eEF2 (*n*=4–8 versus 4–8) with a serum sample (S); relative unit (RU) to that of IL-6Rα^f/f^ (Control). Flow cytometry analyses of gp130 expression in cells isolated from (**g**) liver (*n*=10–12 versus 10–12) and (**h**) EWAT (*n*=10–12 versus 10–13), presented as gp130+ cells in percentage of live CD3+ immune cells (PLIC CD3+) and as amount of gp130 detection in median fluorescence intensity per positive cell (MFI/+Cell). Two-way analysis of variance (ANOVA) with multiple analysis used for statistical analyses (*P<0.05; ***P<0.005 for significant differences between IL-6Rα^f/f^ and IL-6Rα^T-KO^. ^*P*<0.05; ^^*P*<0.01; ^^^*P*<0.005 for significant differences between the two time points of HFD feeding). Error bars presented as s.e.m.

In both of our mouse models, IL-6Rα^f/f^ controls and IL-6Rα^T-KO^ mice, prolonged HFD feeding of 8 extra weeks resulted in a drastic increase of circulating IL-6 levels (Fig. 7a), and in parallel a significant increase of circulating sIL-6Rα levels (Fig. 7b). As levels of circulating IL-6 and sIL-6Rα rose over prolonged HFD feeding, tissue IL-6 content also tended to increase in liver (Fig. 7c) and was significantly elevated in EWAT (Fig. 7d) in mice of both genotypes. In addition to increased concentrations of the ligand IL-6 in circulation and metabolic tissues, a significantly higher amount of sIL-6Rα was also detected in the liver and EWAT of both controls and IL-6Rα^T-KO^ animals at 16 weeks of HFD feeding compared with 8 weeks of HFD feeding (Fig. 7e,f). Collectively, all components of the ligand complex IL-6—sIL-6R, which is critical to promote alternative IL-6 trans-signalling, increased in both circulation and metabolic target tissues upon prolonged HFD feeding.

Next, we aimed to investigate whether the cell-intrinsic responsiveness of T cells to IL-6 trans-signalling might also be altered upon prolonged HFD feeding. To this end, we analysed the expression of the critical IL-6 signalling transducer, gp130, in T cells by flow cytometry at different time points of HFD feeding. Here, the percentage of hepatic gp130-expressing T cells in IL-6Rα^T-KO^ animals was lower than that in IL-6Rα^f/f^ controls at 8 weeks of HFD feeding but increased significantly upon prolonged HFD feeding (Fig. 7g). On the other hand, the staining intensity of gp130 did not differ in hepatic T cells between the two genotypes, but it has significantly increased upon prolonged HFD feeding in mice of both genotypes (Fig. 7g). In EWAT, the percentages of gp130-expressing T cells were comparably higher for both genotypes after prolonged HFD, but the expression intensity per cell decreased in T cells of both genotypes while remaining significantly higher in the T cells of IL-6Rα^T-KO^ animals compared with that of controls (Fig. 7h). Taken together, in addition to altering the stimulus of IL-6 trans-signalling, prolonged HFD feeding also led to altered expression of the T cell-intrinsic mediator of IL-6 trans-signalling, gp130.

**Figure 8:**
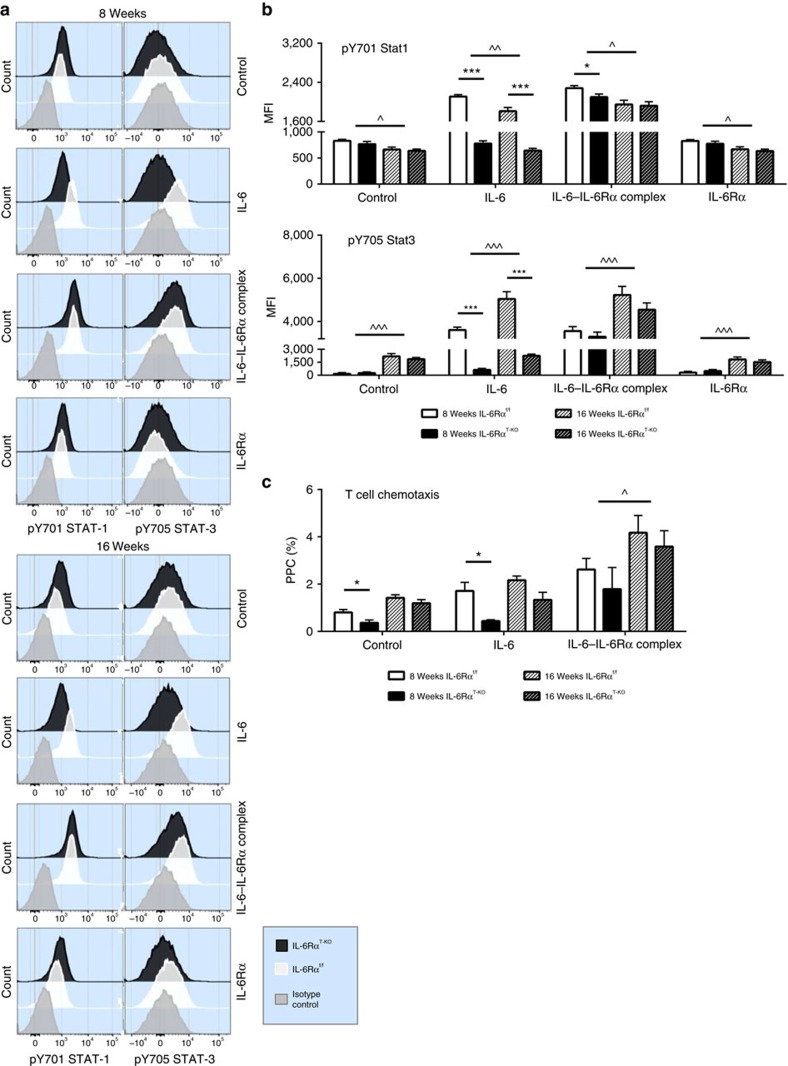
Augmented IL-6 signalling via sIL-6Rα after prolonged HFD feeding normalizes T cell functions in IL-6Rα^T-KO^ (8- and 16-week HFD). (**a**) Compiled representative histograms and (**b**) quantification (MFI) of flow cytometry analyses of stimulated pY701 STAT-1 and pY705 STAT-3 with control (unstimulated), IL-6 (70 ng ml^−1^), IL-6—IL-6Rα complex (200 ng ml^−1^) and soluble IL-6Rα alone (130 ng ml^−1^) in enriched splenic T cell isolation from random-fed IL-6Rα^f/f^ and IL-6Rα^T-KO^ animals at 8 and 16 weeks of HFD feeding (*n*=10–12 versus 10–12). (**c**) Flow cytometry analyses of T cell chemotaxis with control (no treatment), IL-6 (70 ng ml^−1^) and IL-6—IL-6Rα complex (200 ng ml^−1^), presented as percentage of cells pre-chemotaxis (PPC); enriched splenic T cells isolated from random-fed IL-6Rα^f/f^ and IL-6Rα^T-KO^ animals at 8 weeks (*n*=6 versus 4) and 16 weeks (*n*=4 versus 7) of HFD feeding. Two-way analysis of variance (ANOVA) with multiple analysis used for statistical analyses (*P<0.05; ***P<0.005 for significant differences between IL-6Rα^f/f^ and IL-6Rα^T-KO^. ^*P*<0.05; ^^*P*<0.01; ^^^*P*<0.005 for significant differences between the two time points of HFD feeding). Error bars presented as s.e.m.

In light of these observed changes in components of IL-6 trans-signalling upon prolonged HFD feeding, we next directly assessed the responses of T cells to both classical and alternative IL-6 trans-signalling in control and IL-6Rα^T-KO^ mice with purified splenic T cells from control and IL-6Rα^T-KO^ mice after 8 or 16 weeks of HFD feeding. Assessment of IL-6-evoked signal transducer and activator of transcription-1 and -3 (STAT-1 and STAT-3) phosphorylation revealed that while IL-6 clearly promoted phosphorylation of STAT-1 and -3 in T cells of control mice, this response was abolished in T cells of IL-6Rα^T-KO^ mice, further indicating the successful abrogation of classical IL-6 signalling in these mice even upon prolonged HFD feeding (Fig. 8a,b). In contrast, the IL-6—IL-6Rα complex equally promoted STAT-1 and STAT-3 phosphorylation in T cells of both genotypes (Fig. 8a,b). Interestingly, while the ability of trans-signalling to promote STAT-1 phosphorylation did not differ in T cells isolated from mice exposed to HFD feeding for 8 or 16 weeks, T cells isolated from mice exposed to prolonged HFD feeding exhibited significantly enhanced STAT-3 phosphorylation in response to IL-6 trans-signalling (Fig. 8a,b). Collectively, these experiments not only revealed successful abrogation of classical IL-6 signalling in T cells of IL-6Rα^T-KO^ mice, but more importantly also demonstrated that IL-6 trans-signalling could equally activate signal transduction in T cells of both genotypes, which was even partially enhanced upon prolonged HFD feeding.

To investigate whether these observed effects of T cell signalling also translate into functional alterations of T cells, we investigated the ability of classical and trans-signalling of IL-6 to promote chemotaxis of enriched T cells isolated from control and IL-6Rα^T-KO^ mice after 8 weeks and 16 weeks of HFD feeding. Similar to what was observed for STAT-3 phosphorylation, IL-6 promoted chemotaxis of T cells isolated from control but not of the ones from IL-6Rα^T-KO^ mice (Fig. 8c). However, stimulation with the IL-6—IL-6Rα complex equally promoted chemotaxis in T cells from both control and IL-6Rα^T-KO^ mice, and this response was even increased when the animals were exposed to prolonged HFD feeding (Fig. 8c).

Taken together, these experiments indicate that IL-6 trans-signalling can overcome the cellular resistance to classical IL-6 signalling, and that upon prolonged HFD feeding the activator of IL-6 trans-signalling increases both systemically and locally in metabolic target tissues alongside the increased cell-intrinsic propensity of T cells to respond to these signals, at least in part via increased expression of gp130. Functionally, this translates into a profound stimulatory effect on T cells to promote migration and consequently tissue inflammation. Therefore, enhanced IL-6 trans-signalling upon prolonged HFD feeding likely accounts for the abrogation of the protective metabolic effect of inactivated classical IL-6 signalling in T cells of IL-6Rα^T-KO^ mice.

## Discussion

Although IL-6 signalling is crucial for T cell differentiation and function and both IL-6 and T-lymphocytes are important players in inflammation and metabolic disorders^22^,^57^,^58^, the contribution of IL-6 signalling in T cells to the development of obesity-associated chronic low-grade inflammation and insulin resistance had not been previously investigated. In this report, we demonstrate a prominent protection of mice with T cell-restricted IL-6Rα deficiency from diet-induced inflammation and insulin resistance early during the development of obesity. This is consistent with a previous report that IL-6Rα^T-KO^ mice, when treated with ovalbumin and lipopolysaccharide, exhibit unaltered T cell development but demonstrate drastically impaired activation and functions of CD4+ T cells, namely the Th1 and Th17 populations^22^. Similarly, in our HFD-fed obese T cell IL-6Rα-deficient mice with low-grade inflammation, we detect reduced activation and functions of CD4+ T cells, specifically that of IFNγ-secreting Th1 cells, affecting the infiltration of these cells into metabolic tissues and their proinflammatory action of cytokine secretion. Consistent with these data, CD3-specific antibody treatment in obese mice reverses insulin resistance by reducing functional proinflammatory T cells, predominantly the Th1 population^9^.

Due to various practical considerations, most characterization of metabolic phenotypes in genetically modified animal models is usually conducted at one single time point. However, the pathogenesis of obesity-associated inflammation and insulin resistance is a chronic process likely leading to an orchestrated sequential activation of different immune cell types. Therefore, we prolonged the HFD studies to confirm the improved inflammatory and metabolic phenotype in our T cell IL-6Rα-deficient animals at a later stage of obesity development to potentially validate the feasibility of pharmacological interventions by inhibiting T cell IL-6 signalling in the treatment of obesity-associated insulin resistance. Surprisingly, the improved metabolic phenotypes observed at an early point are completely reversed, as the EWAT of IL-6Rα^T-KO^ mice rapidly recruits proinflammatory myeloid cells and acquires an even more inflammatory microenvironment than that of control animals upon prolonged HFD feeding. The altered gene expression in the EWAT of IL-6Rα^T-KO^ mice explains the rapid worsening and even predicts a further deterioration compared with that in control animals. Increased IL-12 release from macrophages and dendritic cells, together with IFNγ, can promote more development of Th1 from CD4+ naive T cells^59^. More RANTES produced by T cells and adipocytes can attract more T cell infiltration^60^. Upregulated iNOS expression can mediate inflammation and tissue damage with adverse metabolic consequences^5^. Moreover, the gene expression of anti-inflammatory cytokine IL-13 (ref. 61) was also significantly lower in the EWAT of IL-6Rα^T-KO^ mice, completing the rapidly worsened inflammatory profile. These drastic changes in the EWAT of IL-6Rα^T-KO^ animals within the extra 8 weeks of HFD regimen may be associated with remodelling of the adipose tissue.

As the main storage depot for fat, adipose tissue remodelling is a constant and dynamic process, responding rapidly to nutrient alterations. Diet-induced obesity promotes adipocyte hypertrophy and pathologically accelerates the remodelling process through tissue inflammation. Being the major mediators of adipose tissue remodelling in obesity, F4/80+ and CD11c+ proinflammatory macrophages surround dying or dead adipocytes in CLSs and assist with the ‘initial necrotic wave'^62^,^63^. According to Strissel *et al*.^63^, the critical point of EWAT remodelling in rodents is ∼16 weeks of HFD feeding, when most death of overly hypertrophied adipocytes occurs and EWAT weighs the least, resulting in reduced cell number and progressively exacerbated tissue inflammation and insulin resistance. Interestingly, after this ‘initial necrotic wave' at 20 weeks of HFD feeding, EWAT is repopulated with more and smaller adipocytes^12^,^63^. Resembling the adipose tissue state immediate post critical point of remodelling, smaller adipocyte size, lower EWAT weight and leptin production (Supplementary Tables 1–4) at week 16 than week 8 are observed in both animal models during our HFD studies. The absence of T cell IL-6 signalling, however, causes a delay in the remodelling process, as the EWAT of IL-6Rα^T-KO^ mice experiences more severe tissue inflammation with more proinflammatory macrophages (Fig. 4) and more pronounced insulin resistance (Fig. 6a) than EWAT of control animals at week 16. These findings reveal the importance of T cell IL-6 signalling in adipose tissue remodelling and support the proposed role for T cells in the remodelling process during HFD-induced obesity development^7^,^9^,^12^.

IL-6 trans-signalling via sIL-6Rα has been reported to direct T cell infiltration^21^ and maintain Th17 functions^64^,^65^ that opens the question of whether enhanced IL-6 trans-signalling in IL-6Rα-deficient T cells could be accounted for the rapid worsening of EWAT inflammation and systemic insulin resistance after the prolonged HFD regimen. To start, the gene expression of IL-17 in liver and EWAT in IL-6Rα^T-KO^ animals is comparable to that in tissues of IL-6Rα^f/f^ controls, and so is the EWAT composition of Th17 with the hepatic composition being even significantly lower (Fig. 4), suggesting a mechanism other than enhanced Th17 activities. In control mice after 16 weeks of HFD feeding, the downregulated IL-6Rα expression in T cells (Supplementary Fig. 1b) appears to be indicative of more inflammation and IL-6 trans-signalling^64^. The significant increase in serum levels as well as tissue content of IL-6 and sIL-6Rα observed from 8 to 16 weeks of HFD feeding in both IL-6Rα^T-KO^ and their control IL-6Rα^f/f^ confers an upregulated IL-6 trans-signalling during prolonged progression of diet-induced obesity (Fig. 7). This augmented IL-6 trans-signalling successfully normalizes the response of IL-6Rα-deficient T cells to IL-6 and the improved mobility in migration (Fig. 8) that not only reveals the strong chemotactic property of both IL-6 and IL-6—IL-6Rα complex but also demonstrates, to our knowledge, for the first time the importance of IL-6 trans-signalling in T cell infiltration. Collectively, these findings argue for an important role of enhanced IL-6 trans-signalling within T cells in the manifestation and maintenance of metabolic inflammation upon prolonged HFD feeding.

From several findings of different tissue immune cells during the course of diet-induced obesity, disappearance and infiltration of specific populations of distinct immune cells have been proposed to be well coordinated and sequential events^2^. Active B cells with functional major histocompatibility complex I and II have been reported to promote T cell activation and infiltration into visceral adipose tissue^66^. The number and responses of B cells in IL-6Rα^T-KO^ animals are also severely affected^22^. In the EWAT of obese IL-6Rα^T-KO^ mice, the proportion of CD19+ B cells within all live CD45+ immune-cell population is significantly reduced compared with that in the EWAT of control mice after 8 weeks of HFD feeding (Supplementary Fig. 8a) as it is for T cells. At 16 weeks however, the proportion of B cells is no longer different (Supplementary Fig. 8b), along which the amount of total T cells, especially the Th1 subtype, rises in the EWAT of IL-6Rα^T-KO^ mice. These observations support the role of B cells in the promotion of T cell infiltration in white adipose tissue and provide a possible compensation that overrides the defective T cell function in IL-6Rα^T-KO^ mice during the progression of tissue inflammation.

As observed from the sequential tissue infiltration by B and T cells, obesity-associated development of inflammation and insulin resistance is a temporal process involving multiple tissues and pathways; hence, the onset of HFD induction should be of critical consideration. For example, using animals fed a HFD (60% Kcal fat) for 8 weeks from 4 to 12 weeks of age, Nishimura *et al*.^7^ showed that infiltrating active CD8+ T cells, not CD4+ T cells, in white adipose tissue drive the recruitment and differentiation of proinflammatory macrophages. In our obese mice fed a similar HFD for 8 weeks from 8 to 16 weeks of age, we have found similar if not more CD4+ T cells in the EWAT as CD8+ T cells with concurrent significant differences in the composition of CD4+ T cells, CD8+ T cells, total macrophages and the proinflammatory CD11c+ macrophages between the two animal models of IL-6Rα^T-KO^ and IL-6Rα^f/f^. After 16 weeks of HFD feeding at 24 weeks of age however, the amount of macrophages is higher in IL-6Rα^T-KO^ mice, while the composition of both CD8+ and CD4+ T cells remain significantly reduced compared with that in the EWAT of control animals, revealing an enhanced macrophage infiltration independent of CD8+ T cells. Focusing on CD4+ T cells, Winer *et al*.^9^ showed that Th2 and not Treg cells are offset by the proinflammatory Th1 cells in tissue inflammation, though they also credited Treg cells for the improvement of glucose and insulin homeostasis following a transient T cell depletion by CD3-specific antibody in obese mice fed a HFD for 10 weeks from 6 weeks of age. The importance of Treg in white adipose tissue is further supported by Feuerer *et al*.^6^, who identified that the Treg cells specific to white adipose tissue are of unique characteristics and the evasion of these cells lifts suppression on effector CD4+ T cells, allowing the progression of excessive inflammation that leads to obesity-associated insulin resistance. Surprisingly, in the EWAT of IL-6Rα^T-KO^ mice, the proportion of Treg cells is significantly reduced at 8 weeks of HFD feeding when both inflammatory and metabolic profiles are more improved. Interestingly, Winer *et al*.^9^ also found the accumulation of the IFNγ-secreting Th1 in obese adipose tissue to be antigen dependent, and a more recent report showed that adipose tissue macrophages act as antigen-presenting cells to stimulate the Th1 production of IFNγ^67^. In our T cell IL-6Rα-deficient animals, we have indeed observed parallelled changes in the EWAT composition of macrophages and IFNγ-secreting Th1 cells between 8 and 16 weeks of HFD feeding. Moreover, these changes seem to be more predominant in EWAT macrophages, supporting the findings by Strissel *et al*.^12^ that T cell infiltration and gene expression of IFNγ in EWAT occur subsequently to macrophage recruitment, although the enrichment of T cells along with gene expression of IFNγ and RANTES was not observed until 20–22 weeks of HFD feeding that started at 5 weeks of age.

Being the primary site for ectopic fat accumulation in obesity, liver develops chronic inflammation and dysregulation of glucose and lipid metabolism that contributes a major share to systemic insulin resistance. To initiate hepatic inflammation, activated resident Kupffer cells and dendritic cells release cytokines to create a proinflammatory microenvironment and chemokines to attract further proinflammatory macrophages and dendritic cells^68^. This then contributes to the development of hepatic steatosis and deterioration of hepatic insulin sensitivity^69^. Though it is known that hepatic T-lymphocytes are mostly activated and that Treg, Th1, Th17 and natural killer T cells are involved in diet-induced alteration of hepatic lipid accretion and nonalcoholic steatohepatitis^68^, the direct participation of T cells in obesity-induced hepatic insulin resistance remains understudied. To date, a human study in obese children first associated increased Th1 cells and their release of IFNγ with nonalcoholic steatohepatitis and insulin resistance^70^. In rodents, it has been found that CD1d knockout mice deficient in natural killer T cells but with fully functional CD8+ T cells are not protected from obesity-associated metabolic abnormalities after prolonged HFD feeding of 26 weeks^71^, indicating a critical role of hepatic CD8+ T cells in this process. Strain differences between C57Bl/6 and BALB/c mice in response to a 24-week HFD regimen differentiate the dominance of Th1 from Th2 in the development of fatty liver, hepatic inflammation and insulin resistance^72^. In our animal models, we have also observed a predominance of CD8+ over CD4+ T cells in the liver. These data are consistent with the correlation of reduced hepatic CD8+ T cells in IL-6Rα^T-KO^ mice, downregulation of proinflammatory gene expression, improvement of insulin sensitivity and reduction in liver fat accumulation after 8 weeks of HFD exposure. More interestingly, prolonged HFD feeding renders the liver of IL-6Rα^T-KO^ animals more insulin resistant, despite the partially retained improvement of hepatic inflammation and reduction in the composition of proinflammatory myeloid cells, Th1 and Th17, as more total CD8+ and CD4+ T cells are found. These findings support the important role of CD8+ T cells and indicate an undetected CD4+ T cell population in the manifestation of hepatic inflammation and insulin resistance in the absence of T cell IL-6 signalling.

Taken altogether, our studies reveal the complex interplay of infiltrating immune cells in orchestrating obesity-associated tissue-specific and systemic insulin resistance in a time-dependent manner. Compromising functional responses of certain immune-cell populations may delay the onset of obesity-associated chronic low-grade tissue inflammation and development of insulin resistance, but this could eventually be compensated by the activation of T cells via alternative IL-6 trans-signalling leading to prolonged activation of the inflammatory state and metabolic disorders in obesity. With the knowledge of our current findings, neutralizing IL-6 signalling in T cells could still be considered, though as a short-term therapy, during the early development of obesity or in a weight-loss programme as a combinational therapy to enhance the effectiveness of diet and exercise.

## Methods

### Animals

All mice (C57BL/6J background) were housed under controlled temperature (22–24 °C) in 12 h light/dark cycle with water and food *ad libitum*, unless fasting was required for experimental purposes. The 8-week-old male mice were switched to a HFD (60% fat by Kcal, total 5.7 Kcal g^−1^ of diet, ssniff EF acc. D12492 (I) mod. Research Diets Inc.) for 8 or 16 weeks. All studies were approved by the local government authority (Bezirksregierung, Cologne, Germany).

### Generation of IL-6Rα^T-KO^ mice and genotyping

Hemizygous CD4-Cre mice^26^ were crossed with IL-6Rα^f/f^ mice^27^ to obtain double heterozygous mice that were again crossed with IL-6Rα^f/f^ mice to generate the initial IL-6Rα^T-KO^ mice. For diet studies, IL-6Rα^f/f^ and IL-6Rα^T-KO^ mice were bred together to produce ≈50% IL-6Rα^T-KO^ mice and ≈50% IL-6Rα^f/f^ littermates^22^. General genotyping was performed by PCR for IL-6 flox (primers: 5GK12, 5′-CCGCGGGCGATCGCCTAGG-3′; 5IL6E × 3, 5′-CCAGAGGAGCCCAAGCTCTC-3′; 3IL6A, 5′-TAGGGCCCAGTTCCTTTAT-3′) and CD4-Cre (primers: forward, 5′-CCCAACCAACAAGAGCTC3′; reverse, 5′-CCCAGAAATGCCAGATTACG-3′).

### Measurement of blood glucose and serum and plasma factors and HOMA-IR

Blood glucose was measured directly using a glucometer (Contour, Bayer). Plasma or serum clinical biochemistry test was performed by the Department of Clinical Chemistry at the University of Cologne Hospital; insulin and C-peptide were quantified by enzyme-linked immunosorbent assay (ELISA) kits (Crystal Chem). HOMA-IR was calculated as fasting blood glucose (mg dl^−1^) × fasting plasma insulin (μU ml^−1^)/405. Serum leptin and IL-6 levels as well as tissue IL-6 content were measured by ELISA kits (R&D Systems).

### General metabolic phenotyping

Body weights were recorded every 2 weeks during the diet studies. Before killing, mice were fasted for 6 h and anaesthetized with an intraperitoneal injection of ketamine-based rodent cocktail. Whole blood retrieved by direct cardiac puncture was used to measure serum insulin, C-peptide and other factors. Intraperitoneal glucose and insulin tolerance tests (intraperitoneal glucose tolerance test (IPGTT) and intraperitoneal insulin tolerance test (IPITT)) were performed after a fasting period of 6 and 2 h, respectively, before intraperitoneal administration of glucose (1 g kg^−1^) or insulin (0.75 U kg^−1^), followed by blood glucose measurement from the tail vein at 0, 8, 15, 30, 60, 90 and 120 min (IPGTT) and 0, 15, 30, 45, 60 and 90 min (IPITT). Glucose-stimulated insulin secretion was assessed by injecting glucose at 0.5 g kg^−1^ body weight through the tail vein and then measuring blood glucose, plasma insulin and C-peptide at time 0, 2, 5, 15, 30 and 60 min.

### HIEG clamp studies

HIEG clamp studies were conducted based on the established method^28^ but in free-moving mice on HFD for 8 and 16 weeks. Jugular vein cannulation was performed 4–6 days before the free-moving HIEG clamp studies to allow sufficient recovery. Each animal was fasted for 4 h and then placed in the clamp chamber for the duration of the clamp experiment. All infusates used in the experiment were prepared with saline containing 3% plasma obtained from donor mice of the same genotypes in the same diet cohort also fasted for 4 h. A primed-continuous infusion of tracer [3-^3^H]glucose (5 μCi priming at 0.05 μCi min^−1^; Perkin Elmer) started 50 min before time 0, when a basal blood sample (60 μl) was collected. Clamping began with a primed-continuous insulin infusion (60 μU prime at 4 μU per g body weight per min; INSUMAN rapid, Sanofi-Aventis), and blood glucose was measured every 10 min using the B-Glucose Analyzer (Hemocue). An average physiological blood glucose concentration of 140 mg dl^−1^ was maintained by infusion of 20% glucose in saline (DeltaSelect). For the analysis of tissue-specific glucose uptake, a bolus of 10 μCi 2[^14^C]deoxyglucose (2[^14^C]DG; American Radiolabeled Chemicals) was infused at 60 min, and blood samples (15 μl) were collected at 6 time points. During the last 30 min of achieved steady state, blood samples (30 μl) were collected every 10 min for the assessment of steady-state parameters. At the end of the experiment (180 min), mice were killed by cervical dislocation; blood sample, brain, liver, EWAT, skeletal muscle (quadricep) and brown adipose tissue were collected. Plasma samples at basal and steady state were processed for the measurement of [3-^3^H]glucose as previously described^73^. For constructing the decay curve of 2[^14^C]DG to calculate tissue-specific glucose uptake, the six plasma samples were measured directly in scintillation counter. Lysates of brain, EWAT, quadriceps and brown adipose tissue were passed through prepared ion-exchange chromatography columns (AGR1-X8 formate resin, 200–400 mesh dry; Poly-Prep Prefilled Chromatography Columns; Bio-Rad Laboratories) for the separation of 2[^14^C]DG from 2[^14^C]DG-6-phosphate (2[^14^C]DG6P). The glucose uptake in each tissue (nmol × g^−1^ × min^−1^) was calculated from the accumulation of tissue 2[^14^C]DG6P and the disappearance rate of 2[^14^C]DG from plasma.

### Tissue preparation

Immediately after cardiac puncture post cervical dislocation, various tissues were excised, cleaned with phosphate-buffered saline (PBS) and then dried gently with a gauze, weighed and snap frozen in liquid nitrogen to be stored at −80 °C. For livers and EWAT collected, ∼10 mg was homogenized fresh in 1 ml peqGOLD TriFast (peqlab VWR) for RNA extraction, and the rest was powdered in liquid nitrogen and stored at −80 °C for further analysis.

### Real-time PCR

Total RNA was extracted and purified using the RNeasy Mini Kit (Qiagen) and used for complementary DNA (cDNA) synthesis using a reverse transcription PCR kit (Applied Biosystems). Real-time PCR was performed using TaqMan primers (see Supplementary Table 9) and system (Applied Biosystems) with 1:20 diluted cDNA product from the reverse transcription.

### Analysis of hepatic lipids

Hepatic lipids were measured by the CECAD Lipidomics Core Facility as previously published^35^. Briefly, mouse liver tissue was homogenized in water (10 mg tissue per 100 μl) using Precellys 24 Homogenisator (PEQLAB) before quantification of diglycerides and triacylglycerides by thin-layer chromatography and ceramide and cholesterol levels by liquid chromatography coupled to electrospray ionization tandem mass spectometry (LC-ESI-MS/MS).

### Western blot analyses and JNK activity assay

JNK kinase activity assay from frozen liver and EWAT was performed using a kit as described by the manufacture (Cell Signaling, 8794). The following antibodies were used for western blotting: anti-eEF2 (Cell Signaling, 2332, 1:1,000 dilution), anti-panAkt (Cell Signaling, 4685, 1:1,000 dilution), anti-pS473Akt (Cell Signaling, 9271, 1:1,000 dilution), anti-pS63-c-Jun (Cell Signaling, 12598, 1:1,000 dilution), anti-c-Jun (Cell Signaling, 23151:1,000 dilution), anti-JNK (Cell Signaling, 9252, 1:1,000 dilution) and horseradish peroxidase-conjugated anti-mouse (Sigma, A4416, 1:5,000 dilution) and anti-rabbit (Cell Signaling, 7074, 1:2,000 or 1:5,000 dilution) secondary antibodies. Horseradish peroxidase of immunoreactive bands was illuminated by Pierce ECL Western Blotting Substrate (ThermoFisher Scientific) and detected by Fusion Solo S with Fusion software (Vilber Lourmat). Densitometry was quantified using ImageQuant TL software (GE Healthcare Bio-Sciences). Phospho-AKT signal was normalized against eEF2 loading control signal on the same membrane and panAKT signal was detected from the same samples as pAKT, but on a different membrane. Normalized values are represented as fold change over IL-6Ra^f/f^ controls. For the JNK activity assay, JNK and eEF2 protein levels were detected in input controls for each sample. Phospho-c-JUN values are represented as fold change over IL-6Ra^f/f^ controls.

### Tissue dissociation and cell separation and general flow cytometry

Immune cells were isolated from spleen, liver and EWAT using the gentleMACS Octo Dissociator (Miltenyi Biotec, Germany) and purified according to the modified protocols from the manufacturer. In brief, splenic cells were dissociated in PBS and then isolated and purified by 2 cycles of filtration through 40 μm strainers (BD Biosciences) with PBS wash and pelleting by centrifugation for 10 min at 400 × *g* before and after the red blood cell (RBC) lysis of 5 min at room temperature; liver was first mechanically disrupted in warm (37 °C) dissociation buffer containing 500 U ml^−1^ collagenase IV (Sigma) and 150 U ml^−1^ DNase I (AppliChem) before being subjected to a 30 min digestion shaking at 250 r.p.m. in 37 °C, after which most hepatocytes were removed and nonparenchymal cells isolated by 2 cycles of 5 min centrifugation at 50 × *g* in 4 °C and filtration through a 100 μm strainer and then a final centrifugation for 10 min at 350 × *g*, all in the isotonic PBS–EDTA (2 mM)–BSA (0.5%) buffer (PEB); further separation and purification of hepatic nonparenchymal cells were carried out by density gradient with Histodenz (Sigma) through brake-free centrifugation for 20 min at 1,500 × *g* followed by RBC lysis and filtration through a 40 μm strainer; EWAT was mechanically disrupted in warm (37 °C) dissociation buffer containing 500 U ml^−1^ collagenase I (Worthington, USA) and digested for 30 min shaking at 250 r.p.m. in 37 °C; isolation and purification of immune cells from the EWAT were then achieved by 3 cycles of PEB wash and centrifugation at 400 × *g* for 10 min as well as a 40 μm filtration after RBC lysis. For detection of IL-17, IL-4 and INFγ by staining, isolated cells were treated for 3 h in 1 ml culture media (1% bovine serum albumin/Dulbecco's modified Eagle's medium (BSA/DMEM)) containing phorbol myristate acetate (40.5 μM) and ionomycin (670 μM) to stimulate cytokine production and brefeldin A (5.3 mM) and monensin (1 mM) to inhibit secretion (1 μl cell stimulation cocktail plus protein transport inhibitors, 00-4970, eBioscience). Before any stimulation or staining, immune cells purified from each tissue in PEB were counted using the flow cytometer MACSQuant VYB (Miltenyi Biotec) and then washed, pelleted and resuspended in staining buffer (5% fetal bovine serum, 0.5 mM EDTA and 0.1% NaN_3_ in PBS) at various densities (1–10 × 10^6^ per ml) depending on the cell viability and staining efficiency. Cell staining and fixation was performed with antibodies based on the conventional method^74^; as negative controls, a separate set of samples were stained with the appropriate isotype control for each antibody used for intracellular staining. Samples were measured on BD FACSCanto II cell analyser equipped with 405, 488 and 633 nm lasers with BD DIVA 7.0 software (BD Biosciences), and data were analysed using BD DIVA 6.0 software. See Supplementary Tables 5 and 6 for detailed information of antibodies/buffers and gating strategies during analysis, respectively.

### Immunohistochemistry

CLSs stained with anti-F4/80 (ab6640; Abcam), along with adipocyte size, were quantified using Fiji ImageJ.

### Enrichment of splenic T cells

Immune cells isolated from the spleen without RBC lysis were treated and stained with the plan T cell isolation kit for mouse (130-095-130; Miltenyi Biotec) and then enriched using autoMACS Pro Separator (Miltenyi Biotec) according to the manual protocol of the manufacturer. Cells were counted using MACSQuant VYB (MQVYB, Miltenyi Biotec). Enrichment was considered successful only when the resulting percentage of CD3+ T cells in total live CD45+ immune-cell population was above 90% (Supplementary Fig. 7a).

### Detection of STAT-1/3 phosphorylation by flow cytometry

Following the manufacturer's guidelines of BD Phosflow Protocol II, the mild alcohol method (BD Biosciences), 10^6^ enriched splenic T cells in 100 μl per well on a conical-bottomed 96-well plate were cultured for 30 min in serum-free (SF) culture medium (RPMI-1640, phenol red free, 1% glutamine and 5% penicillin–streptomycin) before being transferred to another plate with control SF medium (unstimulated), SF media containing freshly prepared 70 ng ml^−1^ IL-6 (406-ML-005, R&D Systems), 200 ng ml^−1^ IL-6—IL-6Rα complex (9038-SR-025, R&D Systems) or 130 ng ml^−1^ sIL-6Rα alone (1830-SR-025, R&D Systems). After stimulation (20 min), cells were pelleted and stained for extracellular markers before lysis, fixation and permeabilization for intracellular staining of pY701 STAT-1 and pY705 STAT-3. Immediately after stimulation, during lysis and long staining periods, cells were kept in the presence of phosphatase inhibitors (PhosSTOP). Samples were measured on MACSQuant10 (MQ10, Miltenyi Biotec). Data were analysed using FlowJo. See Supplementary Tables 7 and 8 for detailed information of antibodies/buffers and gating strategies during analysis, respectively.

### T cell chemotaxis assay

From each sample, 5–6 × 10^5^ enriched splenic T cells were first cultured for 30 min in culture medium (RPMI-1640, phenol red free, 1% glutamine, 5% penicillin–streptomycin and 0.5% fetal calf serum), after which 10^5^ cells were distributed in 96 wells, stained and fixed for pre-migration measurement of T cell subpopulations and expression of IL-6Rα on MQ10, while other portions of 10^5^ cells in 100 μl culture medium were loaded into each 24-well Transwell Permeable Support of 5 μm pore (3421, Corning, USA) for T cell chemotaxis in surface contact with the 600 μl culture medium containing control (only culture medium), freshly prepared 70 ng ml^−1^ IL-6 (406-ML-005, R&D Systems) or 200 ng ml^−1^ IL-6—IL-6Rα complex (9038-SR-025, R&D Systems) as chemokines in the bottom chamber of each well. After incubation (4 h), the support inserts were removed with gentle shaking and then discarded, and migrated cells in the medium from the bottom chamber were collected, pelleted, distributed in 96 wells, stained, fixed and measured on MQ10 as for the pre-migrated cells. Data were analysed using FlowJo. See Supplementary Tables 7 and 8 for detailed information of antibodies/buffers and gating strategies during analysis, respectively.

### Statistical analyses

Statistical analyses were performed by either two-tailed Student's *t*-test to compare data with a single variable or two-way analysis of variance to compare data with two or more variables using Microsoft Excel, JMP-8 program (JMP, Cary, NC, USA) or GraphPad (Prism, USA). The *P* values were considered significant if <0.05. The s.e.m. values are represented in the graphs.

### Data availability

Most data generated or analysed during this study are included in this published article (and its Supplementary Information files). Other related data generated during and/or analysed during the current study are available from the corresponding author on reasonable request.

## Additional information

**How to cite this article:** Xu, E. *et al*. Temporal and tissue-specific requirements for T-lymphocyte IL-6 signalling in obesity-associated inflammation and insulin resistance. *Nat. Commun.* **8,** 14803 doi: 10.1038/ncomms14803 (2017).

**Publisher's note:** Springer Nature remains neutral with regard to jurisdictional claims in published maps and institutional affiliations.

## Supplementary Material

Supplementary Information Supplementary Figures, Supplementary Tables.
