# Supplementary material for: Temporal and tissue-specific requirements for T-lymphocyte IL-6 signalling in obesity-associated inflammation and insulin resistance
Source: Nat Commun. 2017 May 3;8:14803. doi: 10.1038/ncomms14803 (PMC5418621; doi:10.1038/ncomms14803)
Supplement: Supplementary Information — Supplementary Figures, Supplementary Tables. [file ncomms14803-s1.pdf]

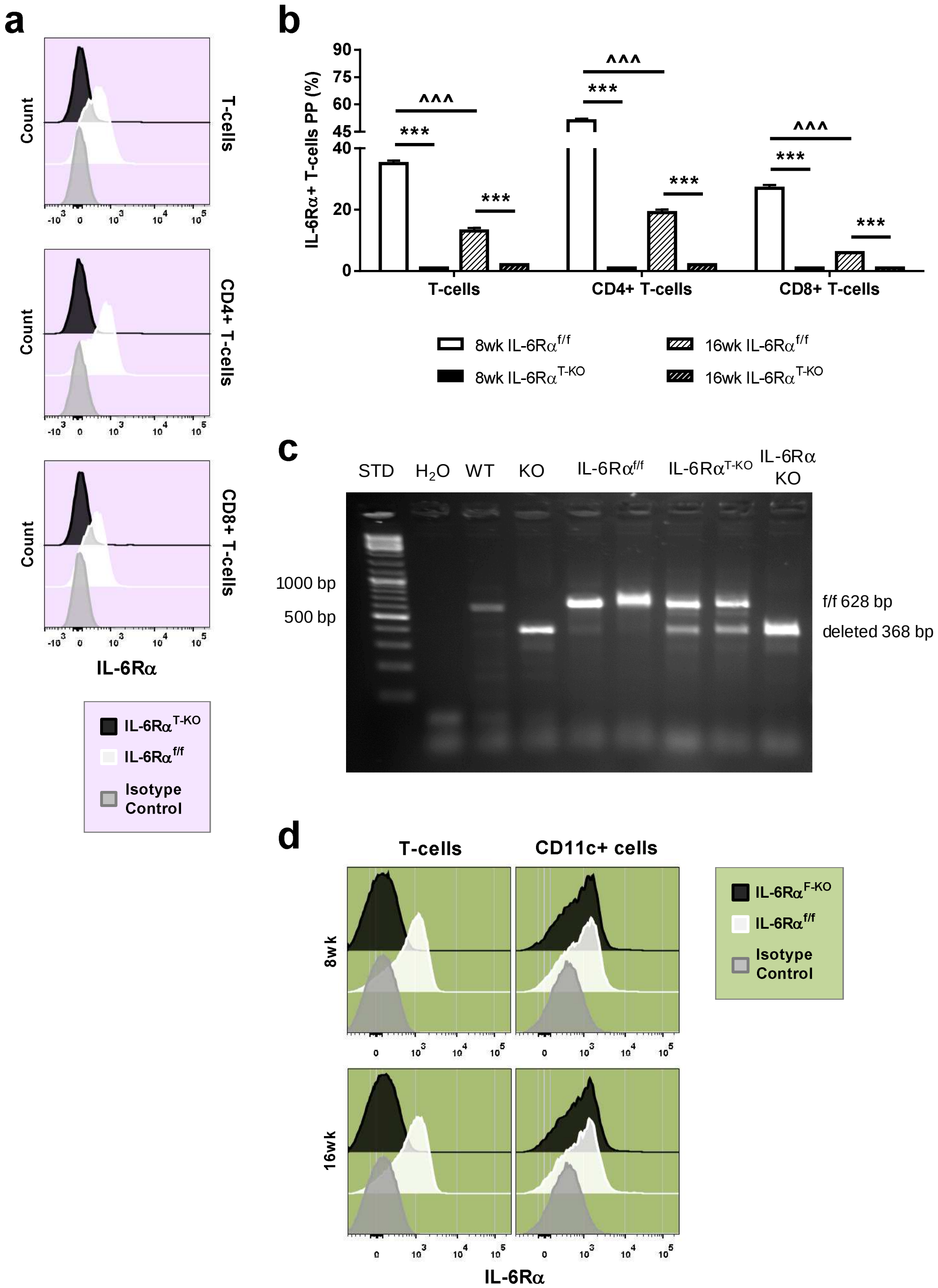

Supplementary Figure 1 | Confirmation of T-cell IL-6Rα deficiency. (**a**) Representative histograms and (**b**) quantification in population percentage (PP) of IL-6Rα+ T-cells from flow cytometry analyses of IL-6Rα in total T-cell population, CD4+ and CD8+ subpopulations enriched and isolated from spleens of control IL-6Rα$^{f/f}$ and IL-6Rα^{T-KO} fed HFD for 8 or 16 weeks; isotype control used as a negative control for staining of IL-6Rα. (**c**) Detection of IL-6Rα deletion by polymerase chain reaction (PCR) for genotyping in splenic tissues from the same control IL-6Rα$^{f/f}$, IL-6Rα^{T-KO} and IL-6Rα KO. (**d**) Representative histograms of flow cytometry analyses of IL-6Rα in total T-cells and CD11c+ cells from spleens of control IL-6Rα$^{f/f}$ and IL-6Rα^{T-KO} fed HFD for 8 or 16 weeks. *** $P < 0.005$ for significant differences between IL-6Rα$^{f/f}$ and IL-6Rα^{T-KO}; isotype control used as a negative control for staining of IL-6Rα. Two-way ANOVA with multiple analysis used for statistical analyses (^^^ $P < 0.005$ for significant differences between the two time points of HFD feeding). Error bars presented as SEM.

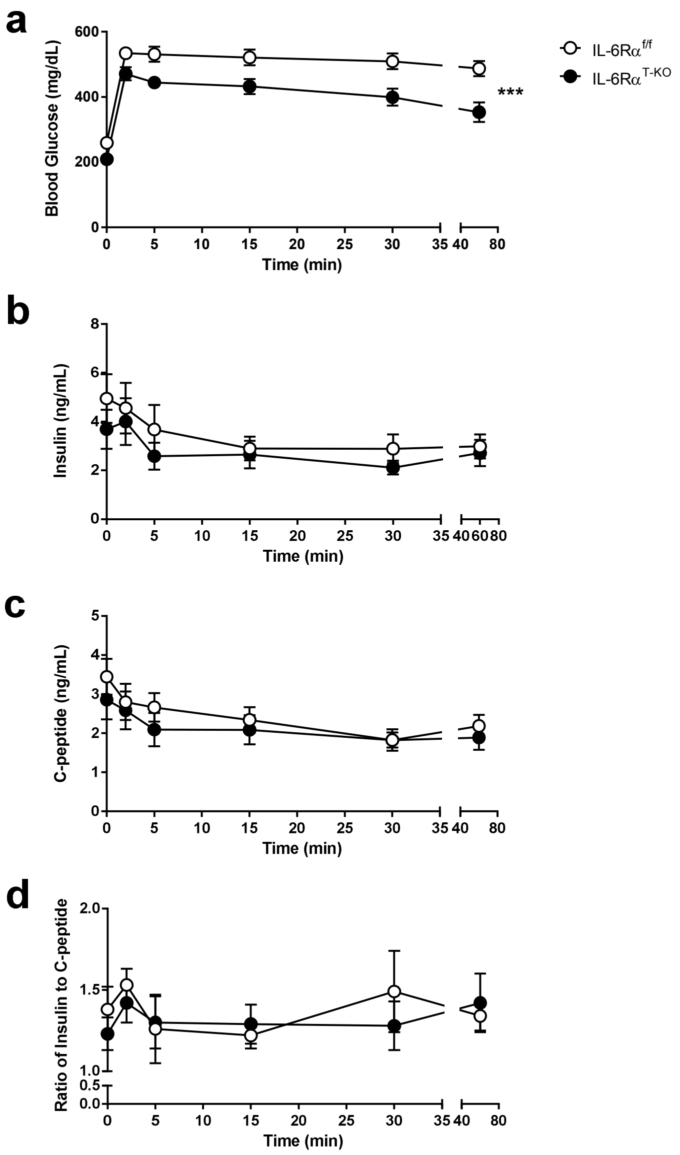

Supplementary Figure 2 | Glucose stimulated insulin secretion (GSIS) in IL-6Rα$^{f/f}$ and IL-6Rα$^{T\text{-}KO}$ (8wk HFD). (**a**) Glucose, (**b**) insulin, (**c**) C-peptide and (**d**) ratio of insulin to C-peptide during GSIS (n = 9). Two-way ANOVA used for statistical analyses (*** *P* < 0.005). Error bars presented as SEM.

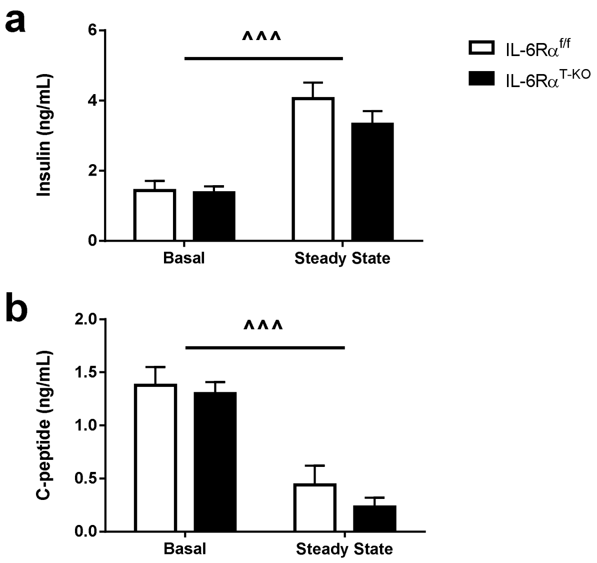

Supplementary Figure 3 | Suppression of insulin secretion during HIEG clamp experiments (8wk HFD). Basal and steady-state plasma levels of (**a**) insulin and (**b**) C-peptide (n = 9). Two-way ANOVA used for statistical analyses (^^^ $P < 0.005$ for difference between basal and steady state). Error bars presented as SEM.

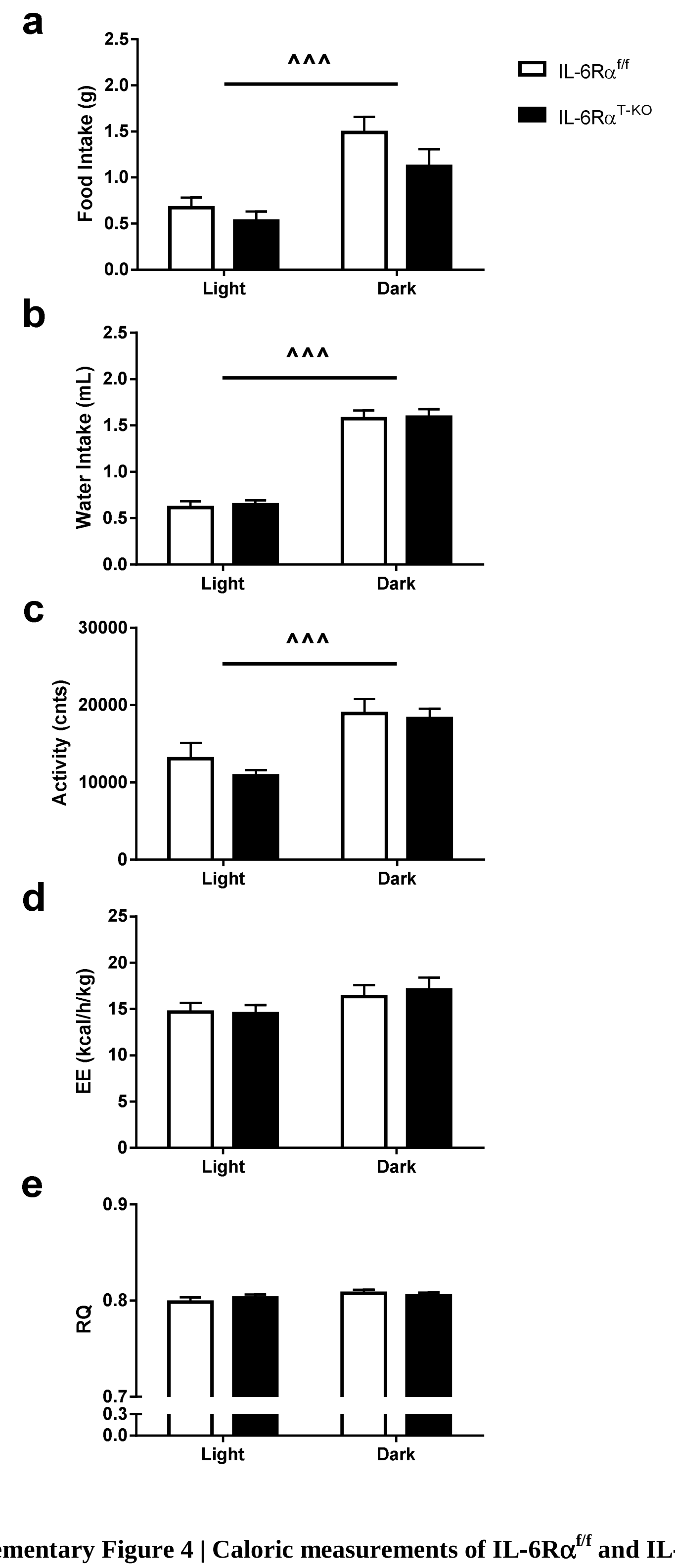

Supplementary Figure 4 | Caloric measurements of IL-6Rα$^{f/f}$ and IL-6Rα^{T-KO} animals (14wk HFD). Caloric measurements of (**a**) cumulative food intake, (**b**) cumulative water intake, (**c**) cumulative activity, (**d**) average energy expenditure (EE) and (**e**) average respiratory quotient (RQ) calculated with respiratory exchange ratio during the light and dark cycles for animals fed HFD for 14 weeks (n = 13 vs. 17). Two-way ANOVA used for statistical analyses (^^^ $P < 0.005$ for significant differences between the two time-points of HFD feeding). Error bars presented as SEM.

a

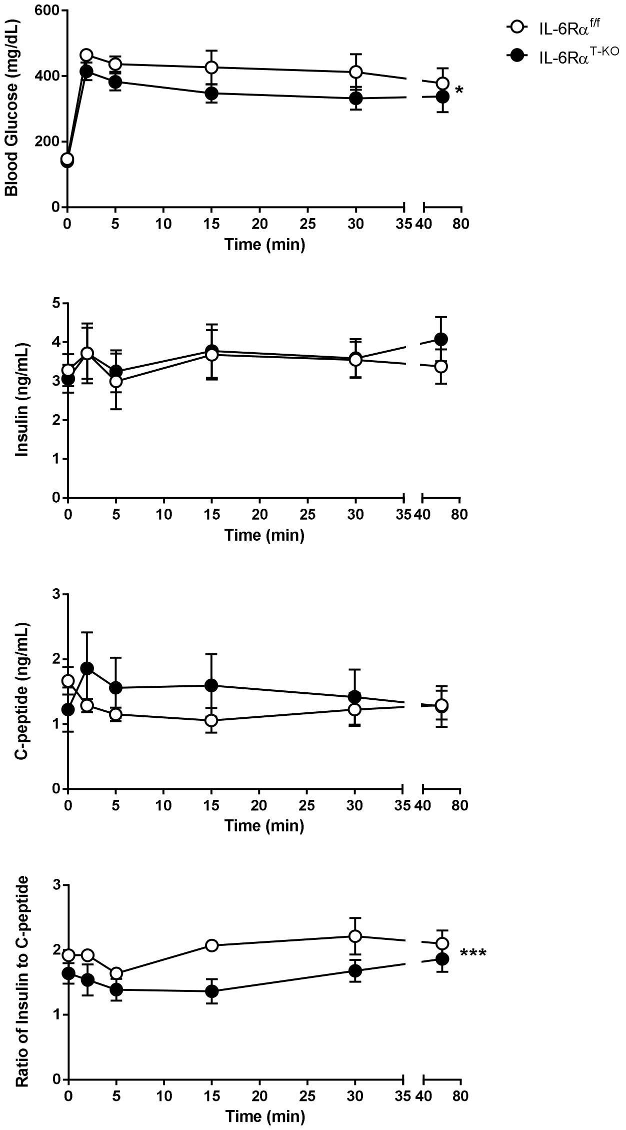

Supplementary Figure 5 | Glucose stimulated insulin secretion (GSIS) in IL-6Rα$^{f/f}$ and IL-6Rα$^{T\text{-}KO}$ (16wk HFD). (**a**) Glucose, (**b**) insulin, (**c**) C-peptide and (**d**) ratio of insulin to C-peptide during GSIS (n = 14 vs. 15). Two-way ANOVA used for statistical analyses (* $P < 0.05$ and *** $P < 0.005$). Error bars presented as SEM.

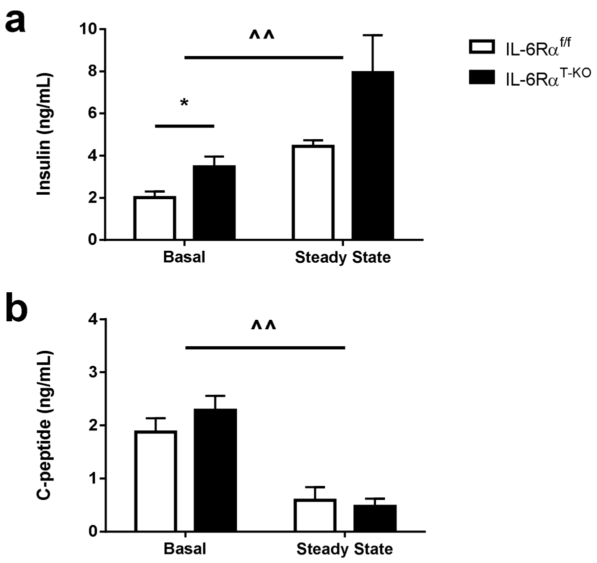

Supplementary Figure 6 | Suppression of insulin secretion and non-esterified fatty acid (NEFA) output during HIEG clamp experiments (16wk HFD). Basal and steady-state plasma levels of (**a**) insulin and (**b**) C-peptide (n = 9). Two-way ANOVA with multiple analysis used for statistical analyses (* $P < 0.05$ for genotypic difference between IL-6Rα$^{f/f}$ and IL-6Rα$^{T\text{-}KO}$ mice; ^^ $P < 0.01$ for difference between basal and steady state). Error bars presented as SEM.

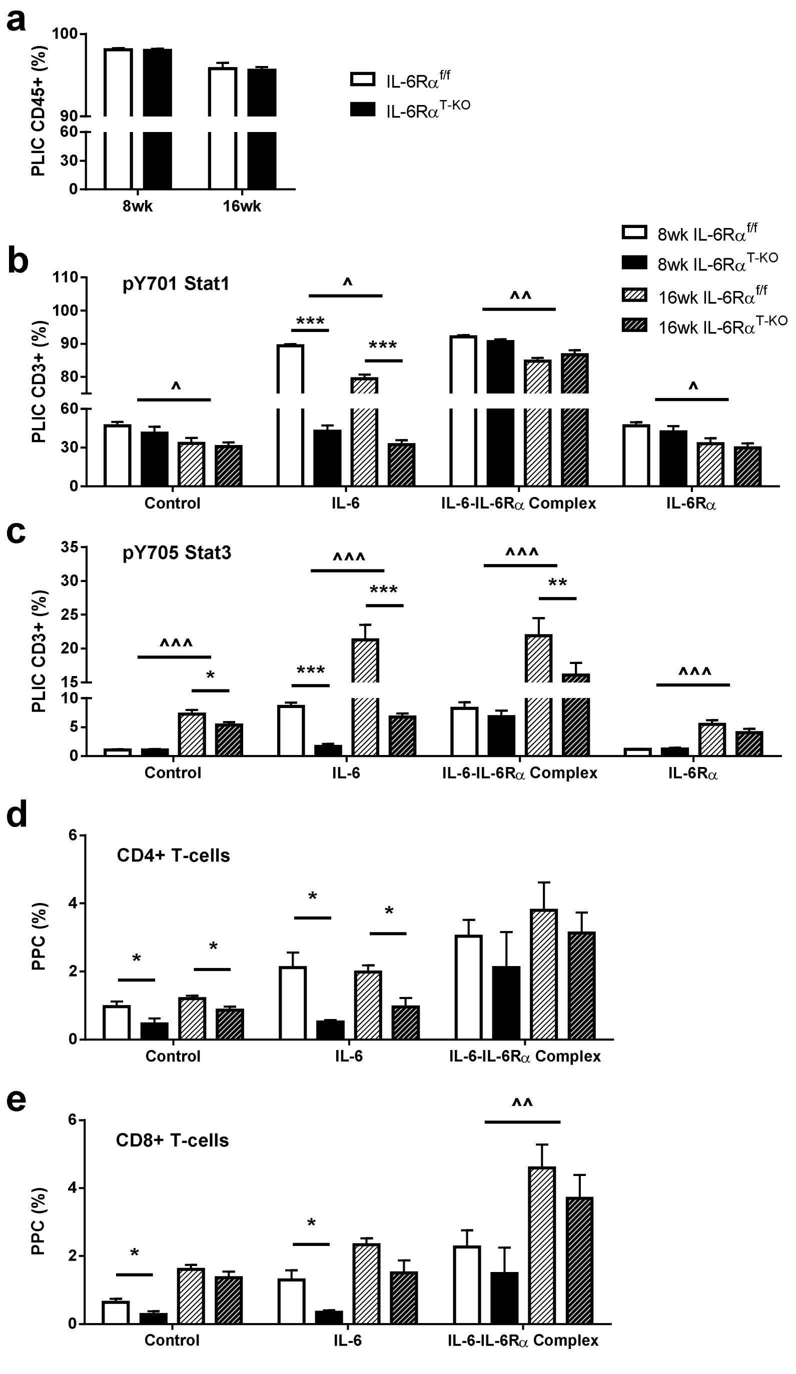

Supplementary Figure 7 | Stimulated phosphorylation of Stat1/3 and migration of T-cells. (**a**) Flow cytometry analyses of enrichment of splenic T-cells isolated from IL-6Rα$^{f/f}$ and IL-6Rα^{T-KO} mice at 8 weeks and 16 weeks of HFD feeding, presented as PLIC CD45+ (n = 4-12 vs. 7-10). Quantification of flow cytometry analyses of activated cells positive for (**b**) pY701 Stat1 and (**c**) pY705 Stat3 with stimulation of control (unstimulated), IL-6 (70 ng/mL), IL-6-IL-6Rα complex (200 ng/mL) and soluble IL-6Rα alone (130 ng/mL) in enriched T-cells (n = 10-12 vs. 10-12), presented as PLIC CD3+. Quantification of flow cytometry analyses of T-cell chemotaxis for (**d**) CD4+ and (**e**) CD8+ T-cells with control (no treatment), IL-6 (70 ng/mL) and IL-6-IL-6Rα complex (200 ng/mL), presented as PPC (n = 4-6 vs. 4-7). Two-way ANOVA with multiple analysis used for statistical analyses (* $P < 0.05$; *** $P < 0.005$ for significant differences between IL-6Rα$^{f/f}$ and IL-6Rα^{T-KO}. ^ $P < 0.05$; ^^ $P < 0.01$; ^^^ $P < 0.005$ for significant differences between the two time-points of HFD feeding). Error bars presented as SEM.

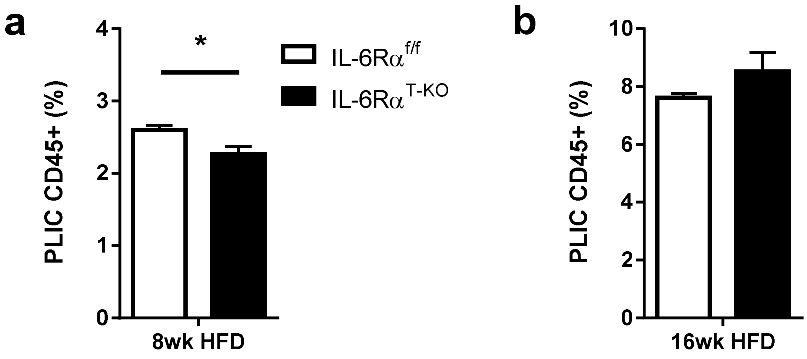

Supplementary Figure 8 | Proportion of B cells in EWAT. Flow cytometry analyses of EWAT composition of B cells in IL-6Rα$^{f/f}$ and IL-6Rα$^{T\text{-}KO}$ mice at (**a**) 8 weeks and (**b**) 16 weeks of HFD feeding, presented as PLIC CD45+ (n = 3 analyzed samples pooled from n = 4-8 animals per sample). Two-tailed T-test used for statistical analyses (* $P < 0.05$). Error bars presented as SEM.

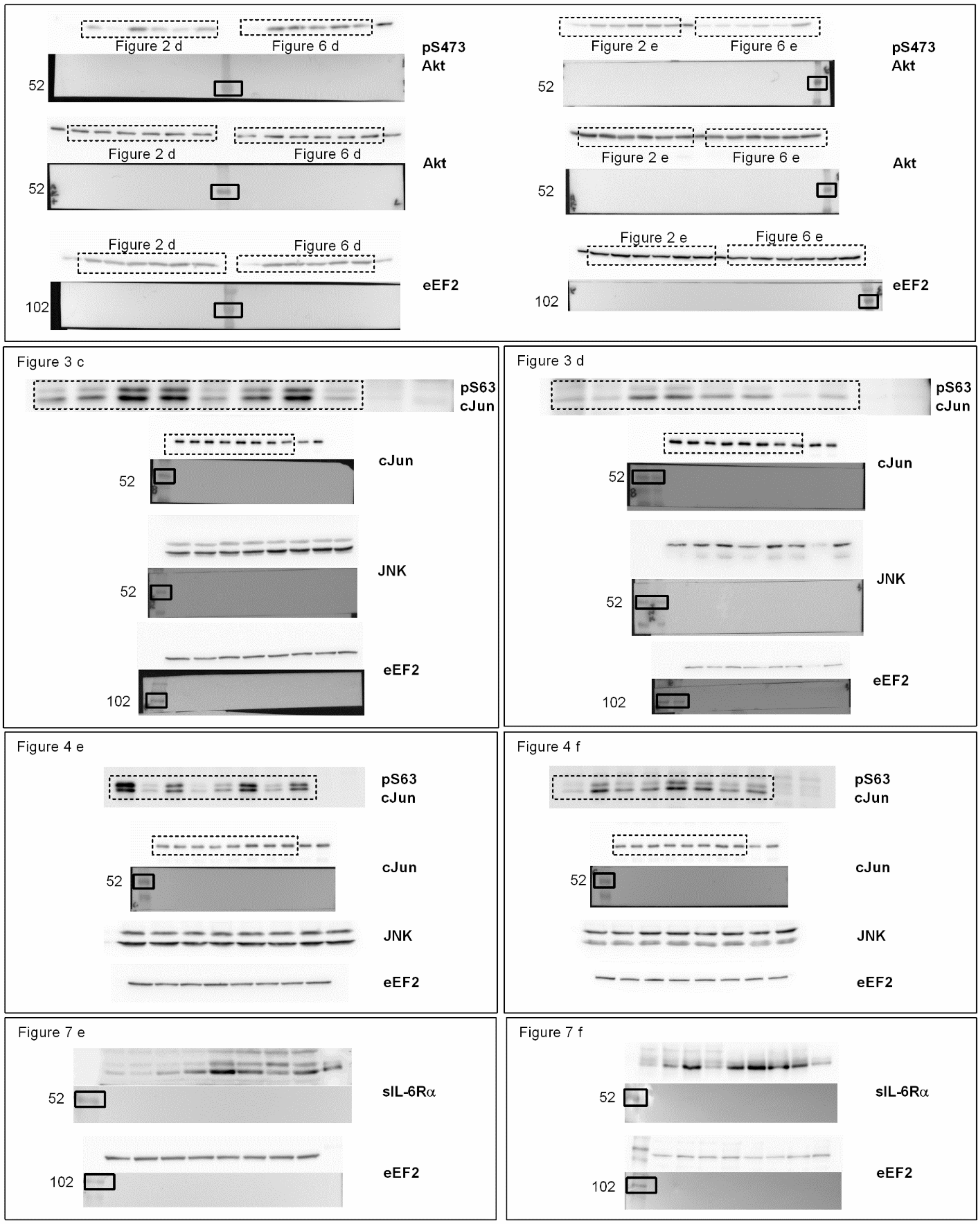

Supplementary Figure 9 | Uncropped scans of the Western blots and molecular weight marker. We provide two images for each blot. The image on top corresponds to the immunoreactive bands and the one on the bottom corresponds to the molecular weight marker of the same membrane (which requires visible light).

Supplementary Table 1				
8wk	Weight (g)		Percentage of BW (%)	
Genotype	*IL6R*$\alpha^{f/f}$	*IL6R*$\alpha^{T\text{-}KO}$	*IL6R*$\alpha^{f/f}$	*IL6R*$\alpha^{T\text{-}KO}$
BW	43.5 ± 1.0	38.7 ± 0.9 ***		
Liver	2.71 ± 0.16	1.80 ± 0.08 ***	6.26 ± 0.39	4.67 ± 0.15 ***
EWAT	2.57 ± 0.05	2.04 ± 0.08 ***	5.86 ± 0.17	5.32 ± 0.20 *
PWAT	1.48 ± 0.08	1.30 ± 0.16	3.24 ± 0.12	2.95 ± 0.29
ScWAT	2.23 ± 0.11	1.83 ± 0.18	4.89 ± 0.23	4.21 ± 0.36
Gastroc	0.31 ± 0.01	0.33 ± 0.01	0.69 ± 0.02	0.79 ± 0.03 *
BAT	0.37 ± 0.02	0.27 ± 0.01 ***	0.85 ± 0.05	0.70 ± 0.03 *

Data are presented as Mean ± SEM, n = 6-32 (* $P < 0.05$, *** $P < 0.005$ by 2-tailed T-tests)

Supplementary Table 2		
8wk	*Genotype*	
Serum Factors	$IL6R\alpha^{f/f}$	$IL6R\alpha^{T\text{-}KO}$
Total Protein (g/dL)	50 ± 2	49 ± 2
Albumin (g/dL)	30 ± 1	28 ± 1 *
Calcium (mg/dL)	2.02 ± 0.04	2.04 ± 0.04
Phosphate (mg/dL)	2.8 ± 0.2	2.9 ± 0.2
AST (U/L)	97 ± 5	79 ± 4 **
ALT (U/L)	64 ± 7	35 ± 3 ***
ALK (U/L)	75 ± 2	73 ± 3
Creatine (mg/dL)	1.5 ± 0.4	1.2 ± 0.3
Leptin (ng/mL)	70 ± 4	63 ± 4
p-Amylase (U/L)	2843 ± 84	2934 ± 99
Lipase (U/L)	34 ± 2	36 ± 2
ChE (kU/L)	6.5 ± 0.2	5.6 ± 0.2 ***
TG (mg/dL)	61 ± 7	68 ± 7
Cholesterol (mg/dL)	186 ± 5	168 ± 6 *
HDL-Cholesterol (mg/dL)	142 ± 2	127 ± 4 ***
LDL-Cholesterol (mg/dL)	32 ± 4	30 ± 5

Data are presented as Mean ± SEM, n = 10-20 (* P < 0.05, ** P < 0.01, *** P < 0.005 by 2-tailed T-tests)

Supplementary Table 3				
16wk	Weight (g)		Percentage of BW (%)	
Genotype	*IL6R*$\alpha^{f/f}$	*IL6R*$\alpha^{T\text{-}KO}$	*IL6R*$\alpha^{f/f}$	*IL6R*$\alpha^{T\text{-}KO}$
BW	48.9 ± 1.1	47.1 ± 1.1		
Liver	2.94 ± 0.20	2.69 ± 0.18	5.94 ± 0.35	5.59 ± 0.30
EWAT	1.74 ± 0.06	1.76 ± 0.05	3.60 ± 0.14	3.81 ± 0.16
PWAT	1.68 ± 0.08	1.46 ± 0.12	3.72 ± 0.12	3.18 ± 0.16 *
ScWAT	2.68 ± 0.13	2.42 ± 0.13	5.49 ± 0.19	5.13 ± 0.15
Gastroc	0.32 ± 0.01	0.34 ± 0.01	0.69 ± 0.03	0.73 ± 0.02
BAT	0.31 ± 0.01	0.25 ± 0.01 **	0.64 ± 0.03	0.53 ± 0.02 **

Data are presented as Mean ± SEM, n = 9-33 (* $P < 0.05$, ** $P < 0.01$ by 2-tailed T-tests)

Supplementary Table 4

16wk	*Genotype*	
Serum Factors	*IL6R*$\alpha^{f/f}$	*IL6R*$\alpha^{T\text{-}KO}$
Total Protein (g/dL)	43 ± 1	44 ± 1
Albumin (g/dL)	26.9 ± 0.7	28.6 ± 0.5
Calcium (mg/dL)	2.0 ± 0.1	2.1 ± 0.1
Phosphate (mg/dL)	3.7 ± 0.2	3.9 ± 0.3
AST (U/L)	161 ± 13	228 ± 14 ***
ALT (U/L)	128 ± 22	143 ± 22
ALK (U/L)	69 ± 6	78 ± 7
Creatine (mg/dL)	0.10 ± 0.01	0.12 ± 0.02
Leptin (ng/mL)	55 ± 2	54 ± 3
p-Amylase (U/L)	2474 ± 83	2500 ± 87
Lipase (U/L)	28 ± 2	22 ± 1 ***
ChE (kU/L)	7.0 ± 0.2	7.4 ± 0.4
TG (mg/dL)	30 ± 4	35 ± 4
Cholesterol (mg/dL)	174 ± 8	185 ± 4
HDL-Cholesterol (mg/dL)	144 ± 5	156 ± 3
LDL-Cholesterol (mg/dL)	23 ± 4	22 ± 4

Data are presented as Mean ± SEM, n = 12-16 (*** P < 0.005 by 2-tailed T-tests)

Supplementary Table 5		
Antibodies and Buffers	Catalogue #	Manufacturer
LIVE/DEAD Fixable Aqua Dead Cell Stain	L34957	Life Technologies
Purified anti-mouse CD16/32 (FcR)	101302	Biolegend
PerCP anti-mouse CD45	130-094-962	Miltenyi Biotec
FITC anti-mouse CD3ε	100306	Biolegend
APC Vio770 anti-mouse CD4	130-096-608	Miltenyi Biotec
PE Vio770 anti-mouse CD8a	130-097-025	Miltenyi Biotec
APC anti-mouse CD25	102012	Biolegend
PE anti-mouse CD19	130-092-041	Miltenyi Biotec
PE anti-mouse F4/80	123110	Biolegend
PerCP anti-mouse/human CD11b	101230	Biolegend
PE/Cy7 anti-mouse CD11c	117318	Biolegend
APC/Cy7 anti-mouse CD14	123318	Biolegend
FITC anti-mouse CD206 (MMR)	141704	Biolegend
APC anti-mouse DC Marker (33D1; DCIR2)	124914	Biolegend
PE anti-mouse/rat CD126 (IL-6Rα)	115806	Biolegend
PE anti-mouse FOXP3	126404	Biolegend
APC anti-mouse IFN-γ	505810	Biolegend
PE/Cy7 anti-mouse IL-4	504118	Biolegend
PE anti-mouse IL-17A	506904	Biolegend
PE rat IgG2b, κ Isotype Control	400607	Biolegend
APC rat IgG1, κ Isotype Control	400411	Biolegend
PE/Cy7 rat IgG1, κ Isotype Control	400415	Biolegend
PE rat IgG1, κ Isotype Control	400407	Biolegend
FOXP3 Fix/Perm Buffer Set	421403	Biolegend
Fixation Medium (Medium A)	GAS001S100	ThermoFisher Scientific

Supplementary Table 6		
Tissue	Target Population	Gating Strategy
Liver and EWAT	T-cells	Immune cells / Singlets / Aqua- (dead-cell stain negative) CD45+ / CD3+
	CD8+ T-cells	Immune cells / Singlets / Aqua- (dead-cell stain negative) CD45+ / CD3+ / CD8+ CD4-
	CD4+ T-cells	Immune cells / Singlets / Aqua- (dead-cell stain negative) CD45+ / CD3+ / CD4+ CD8-
	CD25+ Treg cells	Immune cells / Singlets / Aqua- (dead-cell stain negative) CD45+ / CD3+ / CD4+ CD8- / Foxp3+ CD25+
	Th1 cells	Immune cells / Singlets / Aqua- (dead-cell stain negative) CD45+ / CD3+ / CD4+ / NOT IL-17+ IL-2+ / IFN-γ+
	Th17 cells	Immune cells / Singlets / Aqua- (dead-cell stain negative) CD45+ / CD3+ / CD4+ CD8- / NOT IFN-γ+ IL-2+ / IL-17+
	Th2 cells	Immune cells / Singlets / Aqua- (dead-cell stain negative) CD45+ / CD3+ / CD4+ CD8- / NOT IFN-γ+ IL-17+ / IL-2+
	CD11c+ cells	Immune cells / Singlets / Aqua- (dead-cell stain negative) / CD11c+
	F4/80+ cells	Immune cells / Singlets / Aqua- (dead-cell stain negative) / F4/80+
	MΦ (macrophages)	Immune cells / Singlets / Aqua- (dead-cell stain negative) / F4/80+ / CD14+ CD11b+ / DC-
	CD11c+ MΦ	Immune cells / Singlets / Aqua- (dead-cell stain negative) / F4/80+ / CD14+ CD11b+ / DC- / CD11c+
	F4/80+ DC	Immune cells / Singlets / Aqua- (dead-cell stain negative) / F4/80+ / DC+
	B-cells	Immune cells / Singlets / Aqua- (dead-cell stain negative) CD45+ / CD3- CD19+
Spleen	IL-6Rα+ cells	Lymphocytes / Singlets / CD45+ Aqua- (dead-cell stain negative) / CD3+ (CD4+ CD8- or CD4- CD8+) / IL-6Rα+

Supplementary Table 7		
Antibodies and Buffers	Catalogue #	Manufacturer
LIVE/DEAD Fixable Aqua Dead Cell Stain	L34957	Life Technologies
Purified anti-mouse CD16/32 (FcR)	101302	Biolegend
PE anti-mouse CD3	555275	BD Biosciences
PerCP anti-mouse CD4	553052	BD Biosciences
Alexa 647 anti-Stat3 (pY705)	557815	BD Biosciences
Alexa 488 anti-Stat1 (pY701)	612596	BD Biosciences
PerCP anti-mouse CD45	130-102-469	Miltenyi Biotec
FITC anti-mouse CD3ε	100306	Biolegend
BV421 anti-mouse CD4	562891	BD Biosciences
APC anti-mouse CD8a	130-109-248	Miltenyi Biotec
PE anti-mouse/rat CD126 (IL-6Rα)	115806	Biolegend
Alexa 647 mouse IgG2a, κ Isotype Control	558053	BD Biosciences
Alexa 488 mouse IgG2a, κ Isotype Control	558055	BD Biosciences
PE rat IgG2b, κ Isotype Control	400607	Biolegend
BD Phosflow™ Lyse/Fix Buffer (5 x)	558049	BD Biosciences
BD Phosflow™ Perm Buffer II	558052	BD Biosciences
BD Pharmingen™ Stain Buffer (FBS)	554656	BD Biosciences
PhosSTOP (EASYpack)	4906837001	Roche
MACS BSA Stock Solution	130-091-376	Miltenyi Biotec
autoMACS Rinsing Solution	130-091-222	Miltenyi Biotec
IC Fixation Buffer	00-8222	eBioscience

Supplementary Table 8		
Tissue	Target Population	Gating Strategy
Liver and EWAT	T-cells	Lymphocytes / Singlets / CD45+ Aqua- (dead-cell stain negative) / CD3+
	gp130+ T-cells	Lymphocytes / Singlets / CD45+ Aqua- (dead-cell stain negative) / CD3+ / gp130+
	CD4+ T-cells	Lymphocytes / Singlets / CD45+ Aqua- (dead-cell stain negative) / CD3+ / CD4+ CD8-
	CD8+ T-cells	Lymphocytes / Singlets / CD45+ Aqua- (dead-cell stain negative) / CD3+ / CD4- CD8+
Spleen	T-cells	Lymphocytes / Singlets / CD45+ Aqua- (dead-cell stain negative) / CD3+
	pY701 STAT1 T-cells	Lymphocytes / Singlets / CD3+ Aqua- (dead-cell stain negative) / pY701 STAT1+
	pY705 STAT3 T-cells	Lymphocytes / Singlets / CD3+ Aqua- (dead-cell stain negative) / pY705 STAT3+
	CD4+ T-cells	Lymphocytes / Singlets / CD45+ Aqua- (dead-cell stain negative) / CD3+ / CD4+ CD8-
	CD8+ T-cells	Lymphocytes / Singlets / CD45+ Aqua- (dead-cell stain negative) / CD3+ / CD4- CD8+

Supplementary Table 9	
Gene	Life Technologies Catalogue #
Hprt	Mm01545399_m1
Ifng	Mm01168134_m1
Il1b	Mm01336189_m1
Tnf	Mm00443260_g1
Il6	Mm00446190_m1
Il10	Mm00439614_m1
Ccl2	Mm00441242_m1
Il2	Mm00434256_m1
Il4	Mm00445259_m1
Il12a	Mm00434165_m1
Ccl5	Mm01302428_m1
Nos2	Mm00440502_m1
Il13	Mm00434204_m1
Il17a	Mm00439618_m1
Il17f	Mm00521423_m1
Emr1	Mm00802529_m1
Cd4	Mm00442754_m1
Cd8a	Mm01182108_m1
